# Heterochrony, modularity, and the functional evolution of the mechanosensory lateral line canal system of fishes

**DOI:** 10.1186/2041-9139-5-21

**Published:** 2014-06-05

**Authors:** Nathan C Bird, Jacqueline F Webb

**Affiliations:** 1 Current address: Department of Biological Sciences, University of Rhode Island, 120 Flagg Road, Kingston RI 02881, USA

**Keywords:** *Aulonocara*, *Tramitichromis*, Cichlidae, Neuromast, Dermatocranium, Heterochrony, Modularity, Lateral line

## Abstract

**Background:**

The canals of the mechanosensory lateral line system are components of the dermatocranium, and demonstrate phenotypic variation in bony fishes. Widened lateral line canals evolved convergently in a limited number of families of teleost fishes and it had been hypothesized that they evolved from narrow canals via heterochrony and explore modularity in the lateral line system. Two species of cichlids with different canal phenotypes were used to test a hypothesis of heterochrony. Histological material prepared from ontogenetic series of *Aulonocara stuartgranti* (widened canals) and *Tramitichromis* sp. (narrow canals) was analyzed using ANCOVA to determine rates of increase in canal diameter and neuromast size (length, width) and to compare the timing of onset of critical stages in canal morphogenesis (enclosure, ossification).

**Results:**

A faster rate of increase in canal diameter and neuromast width (but not length), and a delay in onset of canal morphogenesis were found in *Aulonocara* relative to *Tramitichromis*. However, rates of increase in canal diameter and neuromast size among canals, among canal portions and among canals segments reveal similar trends within both species.

**Conclusion:**

The evolution of widened lateral line canals is the result of dissociated heterochrony - acceleration in the rate of increase of both canal diameter and neuromast size, and delay in the onset of canal morphogenesis, in *Aulonocara* (widened canals) relative to *Tramitichromis* (narrow canals). Common rates of increase in canal diameter and neuromast size among canal portions in different dermatocranial bones and among canal segments reflect the absence of local heterochronies, and suggest modular integration among canals in each species. Thus, canal and neuromast morphology are more strongly influenced by their identities as features of the lateral line system than by the attributes of the dermatocranial bones in which the canals are found. Rate heterochrony manifested during the larval stage ensures that the widened canal phenotype, known to be associated with benthic prey detection in adult *Aulonocara,* is already present before feeding commences. Heterochrony can likely explain the convergent evolution of widened lateral line canals among diverse taxa. The lateral line system provides a valuable context for novel analyses of the relationship between developmental processes and the evolution of behaviorally and ecologically relevant phenotypes in fishes.

## Background

Heterochrony, evolutionary change in developmental rate or relative timing of developmental events, plays a key role in the transformation of morphology in evolutionary time [1-5] and can play an important role in both the origin and evolutionary diversification of complex phenotypes [6,7]. Heterochrony may occur at the level of the whole organism, or among phenotypic elements (thus defined as local [3] or regional [8] heterochrony) resulting in novel phenotypes [9-11]. Heterochronic change among phenotypic elements may also occur as the result of a combination of both increases (peramorphosis) and decreases (paedomorphosis) in developmental rates or temporal displacements in the onset and/or offset of developmental events among different phenotypic elements [2]. Such a combination of heterochronic shifts has been described as “dissociated heterochrony” [12] or “mosaic heterochrony” [13]. In vertebrates, heterochrony has been identified as an important aspect of the evolution of the skull of fishes, for example, [14-16], amphibians, for example, [17-19], reptiles, for example, [20], birds, for example, [21] and mammals, for example, [22-26].

**Figure 1:**
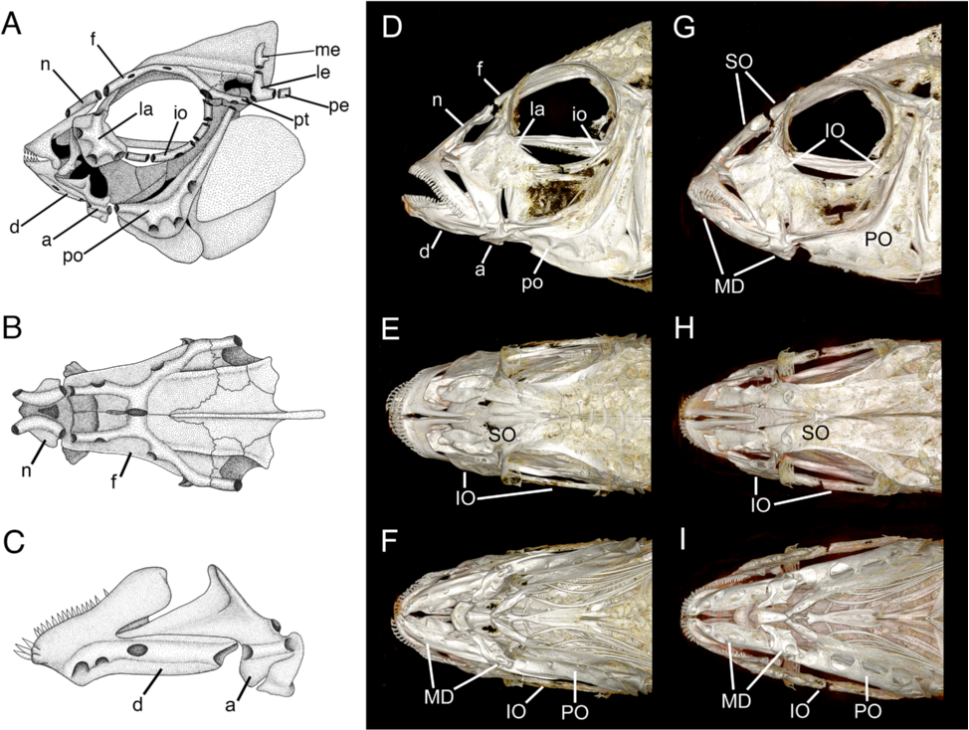
**Lateral line canal system in *Amatitlania nigrofasciata *(A-C), *Tramitichromis *sp. (D-F), and *Aulonocara stuartgranti *(G-I).** Drawings based on a cleared and stained skull of an adult convict cichlid, *A. nigrofasciata***(A-C)** illustrate the different dermatocranial bones that contain the pored lateral line canals. The supraorbital canal (SO) is contained within the nasal and frontal bones and is visible in both lateral and dorsal views **(A, B)**. The mandibular canal (MD) is contained within the dentary and anguloarticular bones, which are visible in lateral view **(C)**. The infraorbital canal (IO) is contained within the lacrimal and infraorbital bones, and the preopercular canal (PO) is contained within the preopercular bone, and is visible in lateral view **(A)**. 3-D reconstructions of μCT data from a *Tramitichromis* sp. (adult, 79 mm SL) in lateral, dorsal and ventral views **(D, E, F)**, and *A. stuartgranti* (adult, 78 mm SL) in lateral, dorsal, and ventral views **(G, H, I)** clearly show larger canal pores in *Aulonocara* (widened lateral line canals), and in the mandibular canal in particular (for example, **I** vs. **F**), when compared to *Tramitichromis* (narrow lateral line canals). a, anguloarticular; d, dentary; f, frontal; io, infraorbital bones; la, lacrimal; le, lateral extrascapular; me, medial extrascapular; n, nasal; pe, posttemporal; po, preoperculum; pt, pterotic. A from [28], reprinted with permission of Academic Press/Elsevier, Inc.; B, C from [29], reprinted with permission of Wiley and Sons, Inc.

The mechanosensory lateral line system is a primitive vertebrate sensory system found in all fishes and in larval and aquatic adult amphibians. In bony fishes it consists of neuromast receptor organs located on the skin and in pored lateral line canals on the head and trunk, reviewed in [27]. On the head, the lateral line canals are embedded within an evolutionarily conserved subset of dermatocranial bones (Figure 1). The supraorbital canal (SO) is contained in the tubular nasal bone, which sits in soft tissue medial to the olfactory sac, and the frontal bone, which forms part of the neurocranial roof and the dorsal edge of the orbit. The infraorbital canal (IO) is found within the series of infraorbital bones (including the lacrimal bone), which border the ventral half of the orbit. The preopercular canal (PO) is found in the preopercular bone, part of the mobile opercular apparatus, which plays an important role in the generation of water flows critical for gill ventilation and feeding. The mandibular canal (MD) is contained within the two bones that compose the lower jaw - the tooth-bearing dentary bone and the anguloarticular bone. The lumen of the MD canal is contiguous with the lumen of the PO canal. The SO, IO and PO canals typically meet just caudal to the eye, continuing caudally as the otic and post-otic canals in the pterotic and extrascapular bones. The post-otic canal continues through the post-temporal and supracleithral bones before continuing into the trunk canal, which is contained within the tubed lateral line scales.

**Figure 2:**
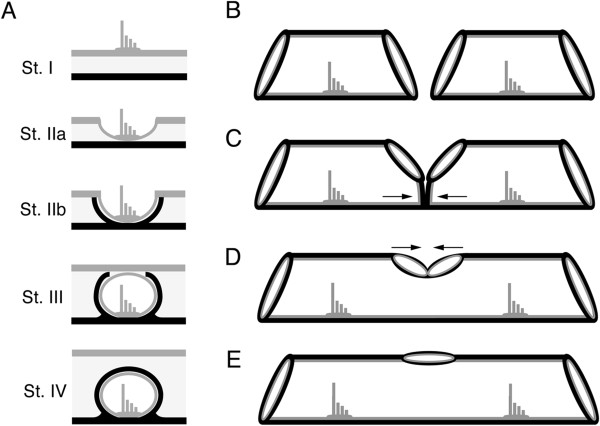
**Neuromast-centered canal morphogenesis and fusion of adjacent canal segments. A)** Stages of neuromast-centered canal morphogenesis (Stages I-IV [29,31]): Stage I – neuromast found in general epithelium, Stage IIa - neuromast sinks into depression, Stage IIb - neuromast in groove with ossified canal walls forming on either side of neuromast, Stage III - neuromast enclosed by soft tissue canal roof, Stage IV - neuromast enclosed in canal and canal roof ossified over neuromast. Canal morphogenesis continues with the gradual fusion of adjacent canal segments. Adjacent canal segments grow toward one another **(B)** and make contact **(C)**. The two adjacent segments fuse **(D)**, leaving a pore between them **(E)**, thus forming a continuous lateral line canal. Black = bone, Gray = general epithelium and neuromasts.

Canal neuromasts are found in stereotyped locations within individual canal segments that compose a canal, a pattern that is the result of neuromast-centered canal morphogenesis [30]. Morphogenesis of individual canal segments occurs in four stages (Figure 2A, Stages I-IV, [29,31]). After differentiation within the epithelium (Stage I) a presumptive canal neuromast sinks into a depression (Stage IIa) and canal walls ossify within the dermis on either side of it (Stage IIb). Next, soft tissue fuses over the neuromast forming a canal segment (Stage III), and, finally, the ossified canal walls extend over the neuromast within the dermis and fuse to form the ossified roof of the canal segment (Stage IV). Adjacent canal segments grow toward one another (Figure 2B,C) and fuse (Figure 2D) leaving a pore between them (Figure 2E), as canal segments become incorporated into underlying dermal bone [32].

Despite the common structural and developmental themes that define canal morphogenesis, several phenotypic variations are found among bony fishes. Five cranial lateral line canal phenotypes are defined - three variations on narrow canals (narrow-simple, narrow-branched, narrow with widened tubules), reduced canals and widened canals [27]. A narrow-simple (“narrow” hereafter) canal phenotype is the most common, and is probably the ancestral condition for bony fishes [27,33]. Narrow-branched and reduced canals have been proposed as the result of positive and negative heterochronic shifts from an ancestral narrow canal phenotype, respectively [33]. Widened canals have also evolved from narrow canals, and are particularly intriguing. They are characterized by a large canal diameter and large canal neuromasts, but also by weak canal roof ossification and large canal pores (presumably the result of a delay or truncation in ossification of the canal roof), suggesting evolution via dissociated heterochrony. Narrow-simple and widened canals are also functionally distinct [34,35]. Widened canals are critical for prey detection [36,37], which provides an important context for understanding of the functional evolution of lateral line canal phenotype.

A test of heterochrony to explain the evolution of widened canals requires the quantitative analysis of the development of closely related species with narrow and widened canal phenotypes. Unfortunately, most fishes with widened canals are inaccessible for study or are particularly difficult to rear in the laboratory [27,38], and pairs of closely related species with narrow and widened canals that are amenable to laboratory study are difficult to identify. However, the speciose African cichlid fishes present unique opportunities for such an analysis. The cranial lateral line canal system of cichlids has been noted in major monographs and taxonomic synopses that describe their cranial osteology [39-41]. Most cichlids have narrow lateral line canals [29,42-44], but a few genera endemic to Lake Tanganyika and Lake Malawi have widened lateral line canals [39,45,46]. A recent comparison of the development of the SO and MD lateral line canals in the Lake Malawi cichlids, *Labeotropheus fuelleborni* and *Metriaclima zebra*, both mbuna (rock dwelling) species with narrow canals, and *Aulonocara baenschi*, a non-mbuna (sand dwelling) species with widened canals, demonstrated that differences in the rate of increase in canal diameter and canal neuromast size can explain the evolution of the widened canal phenotype [47].

This study will use a quantitative analysis of the development of the cranial lateral line canals and canal neuromasts in two closely related sand dwelling (non-mbuna) Lake Malawi cichlids - *Aulonocara stuartgranti* (widened canals) and *Tramitichromis* sp. (narrow canals). Analysis of covariance (ANCOVA) will be used to detect heterochrony in rates of increase in canal diameter and neuromast size (length, width) at the level of the lateral line canal system (SO and MD canals combined), individual canals (SO, MD canals), canal portions in different dermal bones, and individual canal segments in each of the canals. It will then consider how local heterochronies within a species (differences in developmental rates between canals, canal portions, canal segments) reveal independence among modules in the lateral line canal system. It had been shown previously that the two experimental species used here both feed on benthic invertebrate prey in sandy substrates, but use different prey detection strategies [36,37,48]. Thus, this analysis will, for the first time, put heterochrony in the evolution of adaptive phenotypes in the lateral line system in both behavioral and ecological context.

## Methods

**Figure 3:**
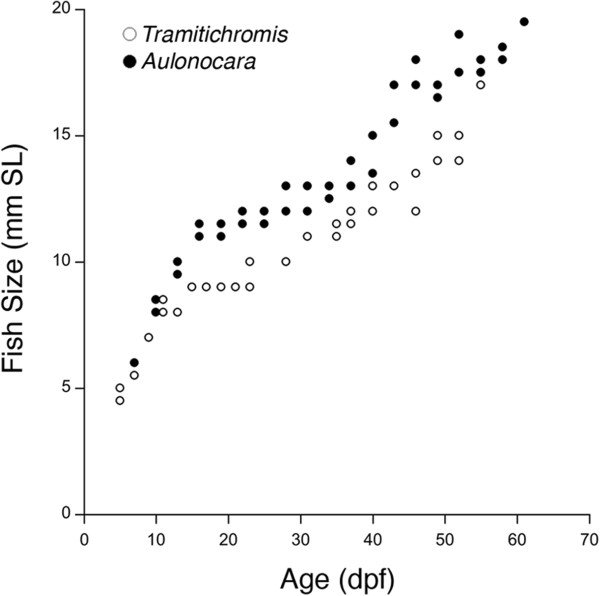
**Growth of *Tramitichromis *sp. (n = 29) and *Aulonocara stuartgranti *(n = 36) prepared histologically.** The yolk sac is absorbed and young are released from the mother's mouth at approximately 20 dpf (10-11 mm SL).

Two species of sand dwelling Lake Malawi cichlids, *Tramitichromis* sp. and *Aulonocara stuartgranti* (referred to by genus throughout), were obtained from Old World Exotic Fish, Inc., (Homestead, FL, USA) or Live Fish Direct (Draper, UT, USA; *Tramitichromis*) and from Bluegrass Aquatics (Louisville, KY, USA; *Aulonocara*). Groups of adults (one male with several females) were maintained in 190 L aquaria in flow-through systems with appropriate mechanical and biological filtration at 80 ± 2°F with 1.0 ± 0.2 ppt salinity (Cichlid Lake Salt, Seachem Laboratories, Inc., Madison, GA, USA) and a 12:12 light cycle. Fish were fed commercial pellet food (New Life Spectrum Cichlid Formula, New Life International, Homestead, FL, USA) one to two times daily. Several days after a brood was noticed (female with expanded buccal cavity), newly hatched fry were removed from the mouth of brooding females and maintained in round-bottom flasks submerged in small tanks and supplied with constant water flow in an AHAB multi-tank flow through system (Aquatic Habitats Inc., Apopka, FL, USA). After yolk absorption, free-swimming fry were allowed to swim out of the flasks into the small tanks and were fed plankton pellets (Hikari^®^ Middle Larval Stage Plankton), then flake food (equal parts egg yolk, earthworm, and Spirulina flakes, Pentair Aquatic Eco-Systems, Inc., Apopka, FL, USA). *Aulonocara* from two broods (AuHb-B017 and AuHb-B021) were sampled every two to three days (6 to 61 days post-fertilization (dpf) and 6 to 112 dpf, respectively) and *Tramitichromis* from a single brood (TRA-B002) were sampled every two to three days (5 to 55 days dpf) to generate ontogenetic series for histological analysis (Figure 3). Fish were anesthetized with MS 222 (ethyl 3-aminobenzoate methanesulfonate; Sigma-Aldrich Co. LLC, St. Louis, Missouri, USA) and fixed in 10% formalin (Sigma F75F) in PBS (phosphate buffered saline, Sigma #P3744), following an approved IACUC protocol.

### Analysis of canal and neuromast development

Histological material was prepared from ontogenetic series of *Aulonocara stuartgranti* (n = 25, 7 to 61 dpf, 6 to 19.5 mm SL) and *Tramitichromis* sp. (n = 20, 5 to 17 mm SL, 5 to 55 dpf). Fish >6 mm SL were decalcified in Cal-Ex Decalcifier (Thermo Fisher Scientific, Waltham, Massachusetts, USA) for two hours (6 to 7.5 mm SL), 3.5 hours (8 to 8.5 mm), or seven to eight hours (>8.5 mm SL), dehydrated in an ascending ethanol and t-butyl alcohol series, and embedded in Paraplast (Thermo Fisher Scientific, Waltham, Massachusetts, USA). Serial transverse sections were cut at 8 μm, mounted on slides subbed with 10% albumin in 0.9% NaCl, and stained with a modified HBQ stain [49].

**Figure 4:**
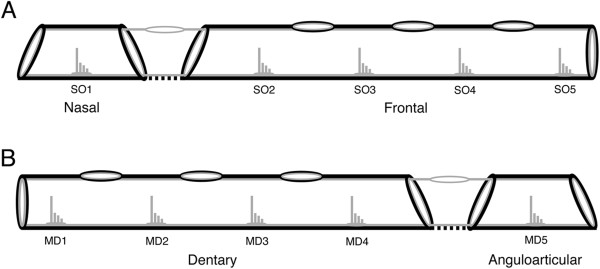
**Distribution of canal neuromasts in the dermatocranial bones containing supraorbital and mandibular lateral line canals.** Schematic representation of the morphology of the supraorbital **(A)** and mandibular **(B)** canals as used for the evaluation of the modular organization of the cranial lateral line canal system. **A)** Five canal neuromasts are contained within the supraorbital canal, which is composed of canal portions in the nasal (SO1) and frontal (SO2 to SO5) bones including five canal segments (SO1 to SO5). **B)** Five canal neuromasts are contained within the mandibular canal, which is composed of canal portions in the dentary (MD1 to MD4) and anguloarticular (MD5) bones including five canal segments (MD1 to MD5). Black = bone, Gray = general epithelium and neuromasts. Note that pores are located between canal segments (see Figure 2), and that bone is lacking between portions at the articulation of adjacent dermatocranial bones.

Analyses of the development of a representative subset of the cranial lateral line canals - the supraorbital (SO) and mandibular (MD) canals and their canal neuromasts (Figures 1 and 4) - were completed in both species. These two canals run rostro-caudally and allow accurate measurements of canal and neuromast dimensions in serial cross-sections. The study of cleared and stained material, μCT images and dried skeletons indicate that these two canals are good representatives of the entire cranial lateral line canal system. Staging of canal development followed the scheme defined by [29] for another cichlid, *Amatitlania nigrofasciata* (=*Archocentrus nigrofasciatus*). Developmental stage (Stage I to IV) was recorded and presence/absence of canal neuromasts was noted in every section allowing the identification of all five canal neuromasts (SO1 to 5, MD1 to 5, Figure 4) and the stage of canal segment development (I-IV) at the location of each neuromast.

Neuromast length was determined for each of the five SO and MD neuromasts in both the right and left canals by counting the number of sections in which neuromast tissue (hair cells and thickened epithelium composed of mantle cells) was present and multiplying the result by section thickness (8 μm, measurement error ± 16 μm). Neuromast width was measured (to the nearest 0.1 μm) at the rostro-caudal midpoint of each canal neuromast by digitally tracing the curve defined by the apical surface of the cells composing the neuromast. Internal canal diameter (defined by surface of ossified canal bone) was measured (to nearest 0.1 μm) in the same section as neuromast width at the point above the neuromast with maximum diameter. Canal diameter could only be measured after canal morphogenesis had commenced, and was thus determined only in those canal segments at Stages II-IV. All measurements were obtained digitally using Spot software (v. 5.0, Diagnostic Instruments, Sterling Heights, MI, USA) on an Olympus BH-2 compound microscope (Olympus America, Center Valley, Pennsylvania, USA) or Zeiss AxioVision software (v 4.6.3) on a Zeiss AxioImager1 compound microscope (Carl Zeiss MicroImaging GmbH, Gottingen, Germany).

Additional ontogenetic series of *Tramitichromis* (n = 15, 13 to 52 dpf, 8 to 14 mm SL) and *Aulonocara* (n = 12, 7 to 112 dpf, 5.5 to 22 mm SL) were enzymatically cleared and stained for cartilage and bone [50] to visualize the overall timing of bone ossification and canal morphogenesis (Stages I-IV) for comparison with histological data. Images of cleared and stained specimens were captured using Spot software (v. 5.0) on a Nikon SMZ1500 dissecting microscope (Nikon Instruments Inc., Melville, New York, USA).

### Statistical analyses

**Figure 5:**
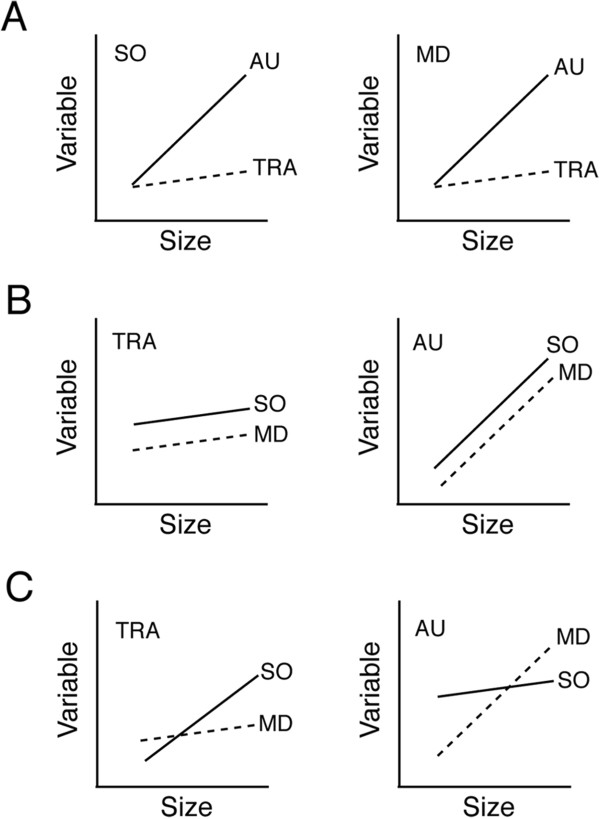
**Expected trends for the analysis of heterochrony and modularity in the cranial lateral line system. A)** Increase in the size of a variable in both experimental species (TRA - *Tramitichromis* sp.; AU - *Aulonocara stuartgranti*) are plotted for two canals (modules; SO - supraorbital; MD - mandibular). Similar trends for both canals (rate increase is faster in *Aulonocara* relative to *Tramitichromis*) is evidence for rate heterochrony. **B)** If both canals (modules) show similar rates within a species, this is evidence for integration among canals. **C)** If the two canals show different rates of increase in a species, this is evidence for independence between canals.

The rates of increase in three variables - canal diameter and neuromast size (length, width) - in the SO and MD canals (combined) were calculated in *Tramitichromis* and *Aulonocara*. Analysis of covariance (ANCOVA) was used to detect differences in rates of increase in canal diameter and neuromast size between species to test a hypothesis of heterochrony. Then, in a second set of ANCOVA’s, rates of increase were compared between species for canals, between canal portions within each canal, and among the five canal segments in each canal, in order to detect local heterochronies (Figure 5A). Left-right mean for each variable was calculated and used in all analyses in order to compensate for asymmetry due to variation in sectioning angle among individuals. All data were found to be normally distributed (Goodness of Fit tests; JMP, v.10.0.2, SAS Institute, Inc, Cary, North Carolina, USA), so data transformation was not required. SL = standard length in mm (fish size) in all analyses including those in supplementary tables. Significance was defined *a priori* as *P* <0.05 for all analyses.

Analyses of developmental rates were carried out with reference to each of the three variables (canal diameter, neuromast length, neuromast width). For a particular variable, if the slopes of the regressions being compared between species (at the level of canal, canal portion, or canal segment) were not statistically different, the data were then tested for mean (elevation) differences using either a Student’s *t*-test (two groups) or Tukey’s honest significant difference (HSD) test (more than two groups). If slopes were different (significant ANCOVA interaction term), the data were subjected to the Johnson-Neyman procedure [51], which identifies the range of x-values (fish size) in which the variable of interest (on y axis) is not statistically different between groups [52], and thus the range in which the parameters are statistically different.

Heterochronic shifts in the onset of developmental events needed to be identified differently. The canal segments that compose a lateral line canal are known to develop asynchronously in other cichlids [29,47], and thus, it was expected that each canal segment in the SO and the MD canals would not necessarily be observed at all four developmental stages (I-IV) among individuals analyzed in this study (as in [47]). Thus, mean fish size at first canal enclosure (Stage III) and canal ossification (Stage IV) for each segment within a canal (SO1 to 5; MD1 to 5), and minimum canal diameter at first canal enclosure (Stage III) and canal ossification (Stage IV) for each segment within a canal, were used to approximate the onset of these processes.

The structural organization of the cranial lateral line canal system (as described above) also lends itself to a consideration of modularity (relative degree of integration and dissociation among modules [6,53-57]). ANCOVA’s were carried out to determine if canal diameter and neuromast size (length, width) in different canals (SO, MD), different portions within a canal, and among segments that compose a canal, demonstrate the same (integration, Figure 5B) or different (dissociation, Figure 5C) developmental rates within each of the two study species.

## Results

**Figure 6:**
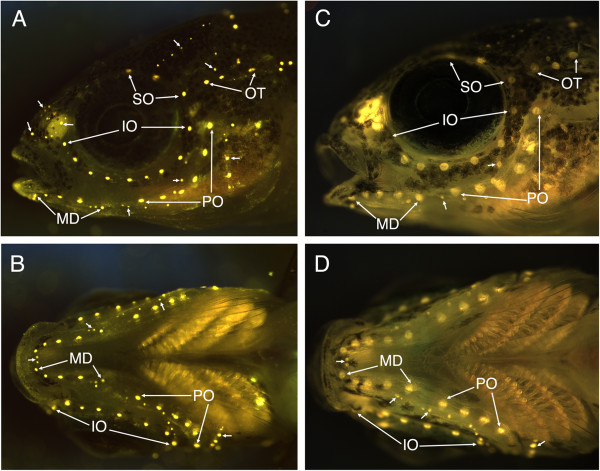
**Fluorescent imaging of neuromasts in *Tramitichromis *sp. and *Aulonocara stuartgranti *using 4-di-2-ASP. A, B)** Lateral and ventral views of *Tramitichromis* sp. (11 mm SL), with arrows pointing to first and last canal neuromasts in the supraorbital (SO), infraorbital (IO), preopercular (PO), mandibular (MD) and otic/post-otic (OT) series. Small arrows point to various groups of superficial neuromasts (see [58] for naming). **C, D)** Lateral and ventral views of *Aulonocara stuartgranti* (11.5 mm SL) labeled as in **A** and **B**. In this individual (the same as visualized with SEM in Figure 7D), the fluorescent label illuminates the entire neuromast (hair cells and surrounding support cells) revealing their diamond shape (as in Figure 7L).

**Figure 7:**
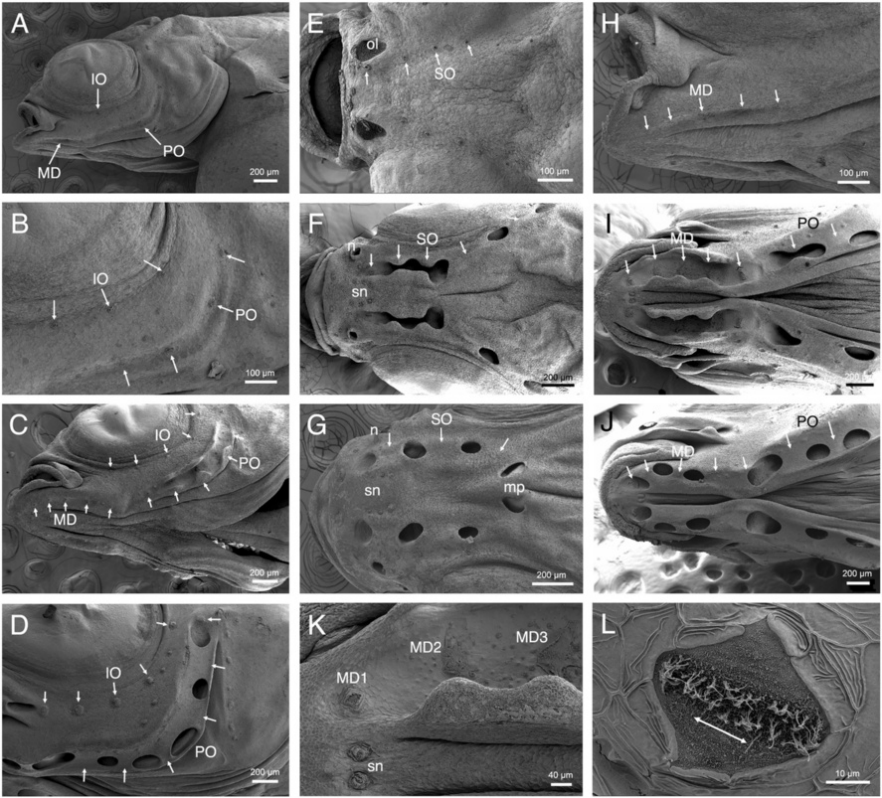
**Neuromasts and canal morphogenesis in *Aulonocara stuartgranti *and *Tramitichromis *sp. visualized with SEM.** All SEM images are of late stage larvae and juveniles of *Aulonocara*, with the exception of **C**, which is of *Tramitichromis*. Arrows indicate presumptive CNs or position of CNs after canal enclosure; rostral to left in all images; see Figure 2 and 8 for definitions of St. 1 to St. 4. **A)** Infraorbital (IO), mandibular (MD) and preopercular (PO) presumptive CNs (canal neuromasts) in yolk sac larva (y, yolk; 7.5 mm SL). **B)** Close-up of A showing IO CNs at St. I, PO CNs at St. IIa. **C)***Tramitichromis* sp. (7.5 mm SL): IO CNs at St. I, MD (MD1 to MD5) at St. I, and PO CNs at St. I or St. IIb. **D)** IO CNs at St. I and PO CNs at St. III/IV (11.5 mm; same individual as in Figure 6C,D). Other neuromasts are small SNs (superficial neuromasts) that remain on skin. **E)** Four supraorbital (SO) CNs; SO1 is medial to olfactory organ (ol) (6.0 mm SL). **F)** SO canal with SO1 at St. I, medial to naris (n), SO2 and SO3 at St. IIb; SO 4 and SO5 at St. III/IV; SNs visible between SO canals (sn; 9.5 mm SL). **G)** SO canal at St. III/IV, arrows indicate SO1 to SO3; two pores caudal to position of right and left SO3 CNs will fuse to form medial pore (mp) (10 mm SL). **H)** Mandibular CNs (MD1 to MD5) at St. IIa (7 mm SL). **I)** MD canal with MD1 at St. IIa, MD2 to 4 at St. IIb, and MD5 at St. IIa. First two PO CNs are at St. IIb and St. III/IV (11 mm SL). **J)** MD and PO canals enclosed (St. III/IV), arrows show MD1 to MD5 and first two PO CNs (12 mm SL). **K)** Close-up of left MD canal in I, showing MD1 at St. IIa, and MD2 and MD3 at St. IIb; small SNs (sn) are round in contrast to diamond-shaped canal neuromasts (11 mm SL). **L)** Close-up of diamond-shaped CN, MD1, showing location of sensory hair cells in sensory strip elongated parallel to physiological orientation of hair cells and canal axis (double-headed arrow; 10 mm SL).

Hatching occurred prior to 7 dpf in both *Tramitichromis* and *Aulonocara*. Yolk sac absorption started by 8 dpf and was complete by 20 to 22 dpf (at 10 to 11 mm SL), when young normally emerge from the mother’s mouth. Neuromasts were evident at hatch, and presumptive canal neuromasts (those that will become enclosed in the lateral line canals) then became distinct in size from superficial neuromasts that remain on the skin (Figures 6 and 7). Canal enclosure started at 8 and 11 mm SL (11 and 16 dpf) in the SO canal, and at 10 and 11 mm SL (23 and 19 dpf) in the MD canal in *Tramitichromis* and *Aulonocara*, respectively (Figure 7).

**Figure 8:**
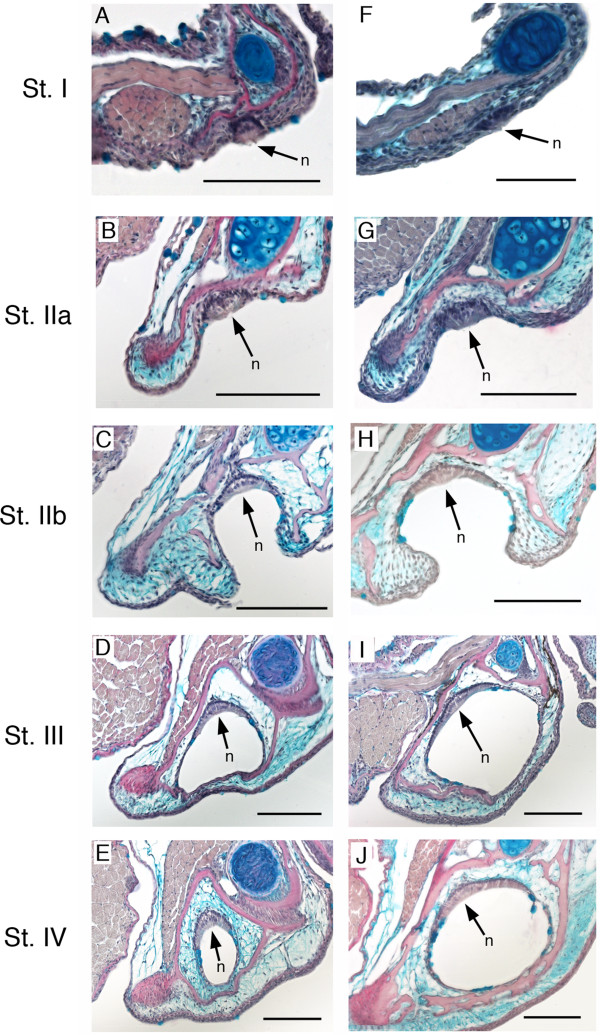
**Stages of neuromast-centered canal morphogenesis in histological sections.** Development of the mandibular canal in *Tramitichromis* sp. (**A**-**E**, narrow lateral line canals), and in *Aulonocara stuartgranti* (**F**-**J**, widened canals). Developmental stages defined by [29] and illustrated schematically in Figure 2A. Pink = bone (stained by direct red), blue = Meckel’s cartilage, mucous cells, or loose connective tissue (stained by Alcian blue). Note that the pattern of canal morphogenesis is the same in both species, but that canal diameter and neuromast width become dramatically larger in *Aulonocara.* N = center of neuromast, indicating location of hair cells (sensory strip). All scale bars = 100 μm.

**Figure 9:**
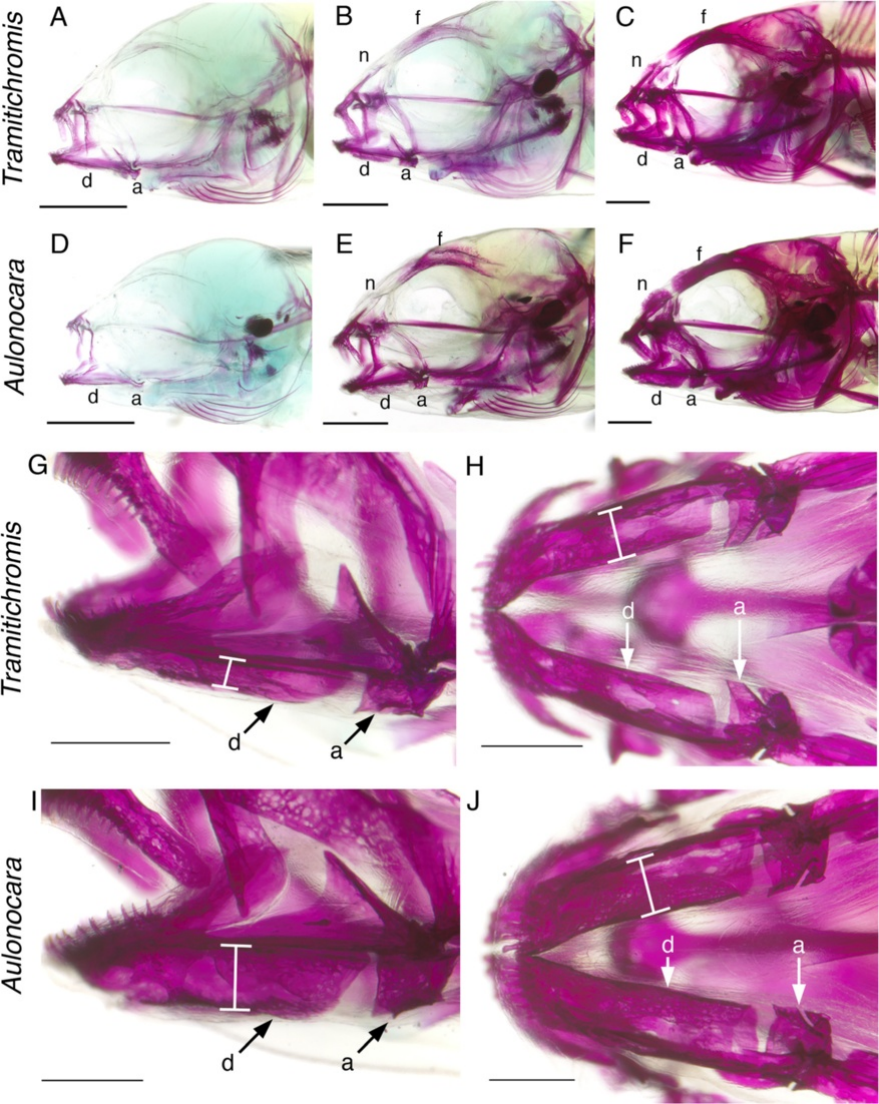
**Bone ossification in cleared and stained larval and juvenile *Tramitichromis *sp. and *Aulonocara stuartgranti*.** Overall developmental sequence is similar in *Tramitichromis* sp. **(A-C)** and *Aulonocara stuartgranti* (**D**-**F**; lateral views, rostral to the left). Early larval development (**A**, **D**; 9 mm SL) showing ossification in jaws and opercular region. The dentary (d) and anguloarticular (a) bones are ossified in both species, but the SO canal is not yet ossified. Older larvae (**B**, **E**; 11.5 mm SL) show ossification spreading to the otic and orbital regions. The frontal (f) and nasal (n) bones are weakly ossified, and the dentary and anguloarticular bones are strongly ossified. Juveniles (**C**, *Tramitichromis*, 14 mm SL; **F**, *Aulonocara*, 16 mm SL) show ossification throughout the cranium. Close ups **(G-J)** of specimens in C and F illustrate the MD canal in lateral **(G, I)** and ventral **(H, J)** views (rostral to the left). Note that the canals are wider in *Aulonocara* than in *Tramitichromis* (white brackets). Scale bars: A-F = 1.0 mm, G-J = 0.5 mm. MD, mandibular canal; SO, supraorbital canal.

Canal neuromast number and distribution (Figure 6), and the process of neuromast-centered canal morphogenesis ([29]; Figures 2, 7, 8) were the same in the two species (see also [47]). As expected from prior descriptions of the lateral line system of cichlids (Figure 1A-C; [29,42,43,47]), neuromast SO1 was found in the portion of the SO canal in the nasal bone, while neuromasts SO2 to 5 were found in the portion of the SO canal in the frontal bone (Figures 1D,E,G,H; 3, 6, 7). Neuromasts MD1 to 4 were found in the portion of the MD canal in the dentary bone, while neuromast MD5 was found in the portion of the MD canal in the anguloarticular bone (Figures 1C,F,I; 3, 7, 9).

The overall pattern and timing of the ossification of cranial bones, including those that contain the lateral line canals, were similar in the two species (Figure 9). Initial bone ossification was apparent in young larvae (9 mm SL) in the upper and lower jaws (including the mandible, which will contain the MD canal) and pharyngeal jaws, the hyoid arch elements and opercular apparatus, and the parasphenoid (Figure 9A,D). Some ossification of the MD canal was visible, in addition to ossification of the dentary and anguloarticular. In older larvae of both species (11.5 mm SL, Figure 9B,E), the size at which young typically emerge from the mother’s mouth, additional ossification was present throughout the cranium, including the frontal bone, and both the SO canal (in the frontal bone) and MD canal (in the mandible). In juveniles of both *Tramitichromis* and *Aulonocara* (Figure 9C,F), ossification was present throughout the cranium, including ossification of all five canal segments composing the SO and MD canals (Figure 9G-J).

### Heterochrony analysis

*Tramitichromis* and *Aulonocara* grew at similar rates (approximately 0.2 mm SL/day, Figure 3), but variation in the rate of increase of SO and MD canal diameter, neuromast length and neuromast width, were evident between species. MD canal diameter (at locations of neuromasts MD 1 to 5) increased at a rate of 2.2 to 3.8 μm/day in *Aulonocara*, but increased at a rate of only 1.6 to 1.8 μm/day in *Tramitichromis*. Canal neuromasts were diamond-shaped in both species (Figure 7L), but appeared to differ in relative length and width between canals and between species (unpublished data). Neuromast length (parallel to canal axis) increased in *Aulonocara* at a rate of 1.4 to 2.0 μm/day, and increased at a comparable rate of 1.5 to 2.1 μm/day in *Tramitichromis*. In contrast, neuromast width (perpendicular to canal axis) increased in *Aulonocara* at a higher rate of 3.3 to 4.2 μm/day, but at a rate of only 1.6 to 1.8 μm/day in *Tramitichromis*.

**Figure 10:**
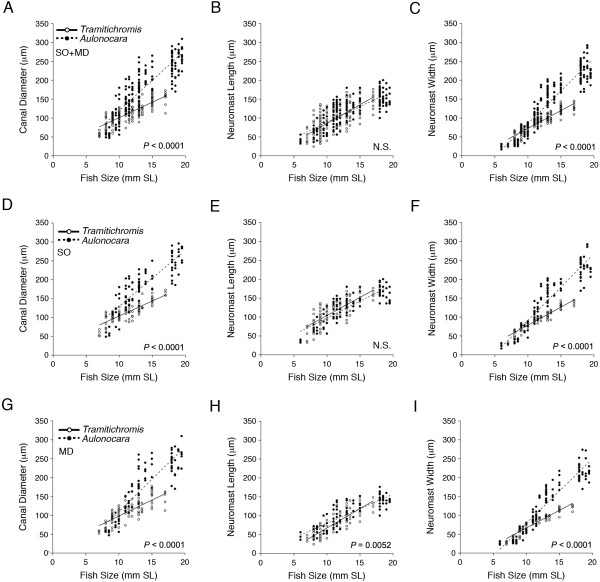
**Comparison of ontogenetic trends in canal diameter and neuromast size in SO and MD canals. A-C)** Comparison of rates of increase in three variables: canal diameter **(A)**, neuromast length **(B)** and neuromast width **(C)** for SO and MD canals combined, in *Tramitichromis* and *Aulonocara*. **D-F)** Comparison of rate of increase in canal diameter **(D)**, neuromast length **(E)** and neuromast width **(F)** for the SO canal in both species. **G-I)** Comparison of rate of increase in canal diameter **(G)**, neuromast length **(H)** and neuromast width **(I)** for the MD canal in both species. See Tables 1 and 2 for results of ANOVA and ANCOVA. Significance level = *P* <0.05; N.S. = no significant difference in rates of increase. MD, mandibular canal; SO, supraorbital canal.

Analyses of the rates of increase in canal diameter and in neuromast length and width in the SO and MD canals (data combined) revealed a higher rate of increase in both canal diameter and neuromast width in *Aulonocara* relative to *Tramitichromis* (Figure 10, Tables 1 and 2). Canal diameter increased approximately 2.0 times faster, and neuromast width increased approximately 1.8 times faster in *Aulonocara* relative to *Tramitichromis* (Table 2, Figure 10A,C)*.* As a result, *Aulonocara* already had wider canals than *Tramitichromis* in young larvae ≥8.6 mm SL, and wider neuromasts in larvae ≥9.1 mm SL (Table 3), which is well before the normal time of release from the mother’s mouth. The rate of increase in neuromast length was not statistically different in the two species (Table 1, Figure 10B).

**Table 1:** ANCOVA for canal diameter and neuromast size (length, width) in the supraorbital and mandibular canals in two species

	***N***	***R***^***2***^	***F***	**d.f.**	***P***
**Canal diameter (combined)**	358	0.83			
Species			175.4131	1, 354	<.0001
SL			732.5361	1, 354	<.0001
Species x SL			77.8231	1, 354	<.0001
**Canal diameter (SO)**	184	0.85			
Species			128.5339	1, 180	<.0001
SL			391.6134	1, 180	<.0001
Species x SL			36.1964	1, 180	<.0001
**Canal diameter (MD)**	174	0.83			
Species			62.1329	1, 170	<.0001
SL			369.4595	1, 170	<.0001
Species x SL			43.2358	1, 170	<.0001
	***N***	***R***^***2***^	***F***	**d.f.**	***P***
**Neuromast length (combined)**	389	0.61			
Species			0.0775	1, 385	0.7809
SL			494.8616	1, 385	<.0001
Species x SL			3.7681	1, 385	0.0530
**Neuromast length (SO)**	194	0.69			
Species			6.0854	1, 190	0.0145
SL			346.0935	1, 190	<.0001
Species x SL			0.4571	1, 190	0.4998
**Neuromast length (MD)**	195	0.73			
Species			5.1958	1, 191	0.0237
SL			405.6425	1, 191	<.0001
Species x SL			7.987	1, 191	0.0052
	***N***	***R***^***2***^	***F***	**d.f.**	***P***
**Neuromast width (combined)**	389	0.89			
Species			179.7582	1, 385	<.0001
SL			1548.252	1, 385	<.0001
Species x SL			135.6519	1, 385	<.0001
**Neuromast width (SO)**	194	0.9			
Species			96.0182	1, 190	<.0001
SL			809.9263	1, 190	<.0001
Species x SL			71.6976	1, 190	<.0001
**Neuromast width (MD)**	195	0.91			
Species			104.6486	1, 191	<.0001
SL			916.1915	1, 191	<.0001
Species x SL			79.312	1, 191	<.0001

Analysis was carried out for supraorbital (SO) and mandibular (MD) canals together (combined) or individually in *Tramitichromis* and *Aulonocara*. SL = Standard length (fish size) in mm. All data were found to be normal. Significance = *P* <0.05. The Johnson-Neyman procedure was used to determine the region of non-significance for fish size for ANCOVAs with significant interaction terms (indicating heterogeneity of slopes, see text and Table 3 for additional details). See Figure 10 and Table 2 for ANOVA results and individual regressions.

**Table 2:** **Results of ANOVA for canal diameter and neuromast size (length, width) in the SO and MD canals in***Tramitichromis***and***Aulonocara*

	***N***	**Regression**	***R***^***2***^	***P***
**Canal diameter (combined)**				
*Tramitichromis*	175	CD = 8.11xSL + 20.24	0.66	<.0001
*Aulonocara*	183	CD = 15.96xSL-39.27	0.76	<.0001
**Canal diameter (SO)**				
*Tramitichromis*	90	CD = 7.9xSL + 25.16	0.71	<.0001
*Aulonocara*	94	CD = 14.81xSL-17.83	0.75	<.0001
**Canal diameter (MD)**				
*Tramitichromis*	85	CD = 8.41xSL + 14.16	0.64	<.0001
*Aulonocara*	89	CD = 17.16xSL-61.74	0.78	<.0001
	***N***	**Regression**	***R***^**2**^	***P***
**Neuromast length (combined)**				
*Tramitichromis*	180	NML = 9.90xSL-12.99	0.51	<.0001
*Aulonocara*	209	NML = 8.31xSL + 5.73	0.65	<.0001
**Neuromast length (SO)**				
*Tramitichromis*	90	NML = 9.57xSL + 8.68	0.62	<.0001
*Aulonocara*	104	NML = 8.90xSL + 8.77	0.71	<.0001
**Neuromast length (MD)**				
*Tramitichromis*	90	NML = 10.23xSL-34.67	0.7	<.0001
*Aulonocara*	105	NML = 7.71xSL + 2.91	0.7	<.0001
	***N***	**Regression**	***R***^**2**^	***P***
**Neuromast width (combined)**				
*Tramitichromis*	180	NMW = 9.44xSL-21.67	0.84	<.0001
*Aulonocara*	209	NMW = 17.38xSL-88.66	0.87	<.0001
**Neuromast width (SO)**				
*Tramitichromis*	90	NMW = 9.56xSL-16.92	0.89	<.0001
*Aulonocara*	104	NMW = 17.66xSL-85.10	0.87	<.0001
**Neuromast width (MD)**				
*Tramitichromis*	90	NMW = 9.31xSL-26.42	0.88	<.0001
*Aulonocara*	105	NMW = 17.08xSL-92.06	0.88	<.0001

Analysis was carried out for supraorbital (SO) and mandibular (MD) canals together (combined) or individually in *Tramitichromis* and *Aulonocara*. SL = standard length (fish size) in mm. Significance level = *P* <0.05. All data were normally distributed. See Figures 10 and 12 and Tables 1 and 5 for results of ANCOVAs. CD, canal diameter; NML, neuromast length; NMW, neuromast width.

**Table 3:** Results of Johnson-Neyman procedure for identifying a region of non-significance in fish size (mm SL) for canal diameter and neuromast shape

		**Variables**	
	**Canal diameter**	**Neuromast length**	**Neuromast width**
**Canals**			
SO Canal	>8.0 mm SL	N/A	>9.4 mm SL
MD Canal	>9.9	<12.6*	>9.4
**Canal Portions**			
Nasal (SO)	>10.3	N/A	>10.0
Frontal (SO)	>7.8	N/A	>9.4
Dentary (MD)	>10.1	<12.5*	>9.5
Anguloarticular (MD)	>10.5	N/A	>9.8
**Canal Segments**			
SO1	>10.3	N/A	>10.0
SO2	N/A	N/A	>10.0
SO3	>8.2	N/A	>10.1
SO4	>12.2	N/A	>11.8
SO5	>9.3	>12.7**	>9.8
MD1	N/A	N/A	>10.3
MD2	>10.9	N/A	>10.0
MD3	>11.3	<11.9*	>10.2
MD4	>11.5	N/A	>10.4
MD5	>10.5	N/A	>9.8

This statistical procedure was carried out for data analyzed at each level in the lateral line system (canal, canal portion, canal segment; see text for additional information) in the supraorbital (SO) and mandibular (MD) canals for which ANCOVAs demonstrated significantly different slopes between species. Numerical values represent the fish size (mm SL) above which *Aulonocara* has wider canal diameters, longer neuromast lengths, and/or wider neuromast widths, except as noted. *fish size (mm SL) below which the variable has a higher value in *Aulonocara* than in *Tramitichromis*. **fish size (mm SL) above which the variable has a higher value in *Tramitichromis* than in *Aulonocara*. N/A indicates that the slopes comparing trends for *Aulonocara* and *Tramitichromis* were not statistically different, so the Johnson-Neyman procedure was not used.

When the SO and MD canals were considered separately, the rates of increase in canal diameter and neuromast width in each canal were also higher in *Aulonocara* than in *Tramitichromis* (Tables 1, 2, Figure 10D,F,G,I). SO and MD canal diameter both increased approximately 2.0 times faster in *Aulonocara* (Table 2, Figure 10D,G) such that *Aulonocara* already had wider SO canals than *Tramitichromis* in small larvae ≥8.0 mm SL and wider MD canals in larvae ≥9.9 mm SL (Table 3). SO and MD neuromast width increased 1.8 to 1.9 times faster in *Aulonocara,* respectively (Table 2, Figure 10F,I), such that *Aulonocara* already had wider SO and MD neuromasts than in *Tramitichromis* in young larvae ≥9.4 mm SL (Table 3). The rate of increase of SO neuromast length was not statistically different between species (Tables 1, 2, Figure 10E), but SO neuromasts were consistently longer in *Aulonocara* than in *Tramitichromis* (Additional file 1: Table S1). In contrast, MD neuromast length increased 1.3 times faster in *Tramitichromis* than in *Aulonocara* (Table 2, Figure 10H), but, MD neuromasts in *Aulonocara* were already longer than those in *Tramitichromis* in small larvae, allowing MD neuromast length *Tramitichromis* to catch up to *Aulonocara* by the time larvae were ≥12.6 mm SL (Table 3).

When the two canal portions in the SO and in the MD canals were considered separately, the rates of increase in canal diameter and in neuromast width were found to be faster in *Aulonocara* than in *Tramitichromis* (Additional file 1: Table S2). Canal diameter increased 1.8 to 2.3 times faster (Additional file 1: Table S3), and neuromast width increased 1.8 to 2.0 times faster, in each of the two portions of the SO and the MD canals (Additional file 1: Table S3). As a result, the two canal portions in the SO and in the MD canals were wider in *Aulonocara* than in *Tramitichromis* in young larvae of >7.8 to 10.5 mm SL and neuromasts were already wider in *Aulonocara* in larvae of >9.4 to 10.0 mm SL (Table 3). The rates of increase in neuromast length in the two portions of the SO and MD canals revealed a rate difference in the neuromasts in the dentary portion of the MD canal (MD1 to MD4; Additional file 1: Table S2). In contrast, the rate of increase in neuromast length was slower (0.74x) in *Aulonocara* than in *Tramitichromis.* The rate of increase in neuromast length in both SO canal portions and in the anguloarticular portion of the MD canal were the same in the two species (Additional file 1: Table S3).

When the five canal segments in the SO (1 to 5) and in the MD (1 to 5) canals were considered separately, rates of increase in canal diameter and neuromast size (length, width) showed inconsistent patterns (Additional file 1: Tables S4-S6). For instance, most but not all MD segments demonstrated a higher rate of increase in canal diameter (Additional file 1: Table S4, S7) and neuromast width in *Aulonocara* (Additional file 1: Table S6, S7). Overall, variability in rate of increase and the size at which the two species demonstrated statistical differences in morphology was more extensive in analyses of individual canal segments (Table 3).

**Figure 11:**
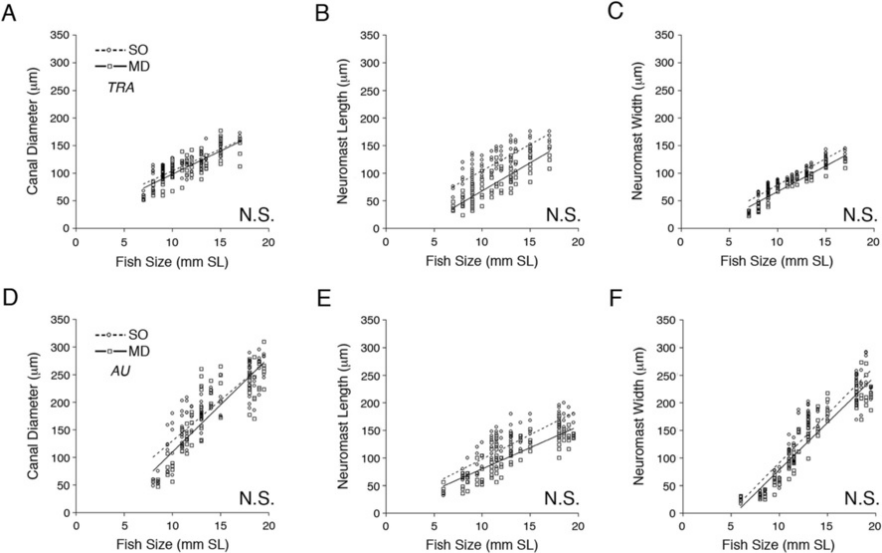
**Ontogenetic trends in canal diameter and neuromast size (length, width) in SO and MD canals. A-C)** Comparison of rates of increase in three variables: canal diameter **(A)**, neuromast length **(B)** and neuromast width **(C)** in *Tramitichromis*. **D-F)** Comparison of rates of increase in three variables: canal diameter **(D)**, neuromast length **(E)** and neuromast width **(F)** in *Aulonocara* derived from histological material. See Tables 2 and 4 for statistical analysis. *P* <0.05 = significant; N.S. = no significant difference in rate of increase in neuromast size between supraorbital (SO) and mandibular (MD) canals.

The analysis of developmental rates for canal diameter, neuromast length and neuromast width within each species at the level of canal, canal portion and canal segment revealed few rate differences that would indicate the presence of local heterochronies within species and thus modularity. Rates of increase in SO and MD canal diameter and neuromast size (length, width) were not statistically different between canals in either species (Tables 2 and 4, Figure 11). The two portions of the SO canal and the MD canal demonstrated similar rates of increase in canal diameter and neuromast size in each species (Additional file 1: Tables S3 and S8). In contrast, the analysis of developmental rates among SO and among MD canal segments revealed some variability, but consistent patterns could not be detected in either species (Additional file 1: Tables S7 and S9).

**Table 4:** **ANCOVA for canal diameter and neuromast size in the SO and MD canals combined in *Tramitichromis *** **and *Aulonocara***

	**N**	**R**^**2**^	**F**	**d.f.**	***P*-value**
**Canal diameter ( *Tramitichromis *)**	175	0.67			
SL			348.0619	1, 171	<.0001
Canal			5.0408	1, 171	0.026
SL x Canal			0.3324	1, 171	0.5650
**Canal diameter ( *Aulonocara *)**	183	0.77			
SL			582.8945	1, 179	<.0001
Canal			5.708	1, 179	0.0179
SL x Canal			3.1312	1, 179	0.0785
	**N**	**R**^**2**^	**F**	**d.f.**	***P*-value**
**Neuromast length ( *Tramitichromis *)**	180	0.74			
SL			338.8333	1, 176	<.0001
Canal			149.0871	1, 176	<.0001
SL x Canal			0.3759	1, 176	0.5406
**Neuromast length ( *Aulonocara *)**	209	0.73			
SL			487.6754	1, 205	<.0001
Canal			54.3751	1, 205	<.0001
SL x Canal			2.4841	1, 205	0.1165
	**N**	**R**^**2**^	**F**	**d.f.**	***P*-value**
**Neuromast width ( *Tramitichromis *)**	180	0.89			
SL			1362.844	1, 176	<.0001
Canal			76.2438	1, 176	<.0001
SL x Canal			0.2324	1, 176	0.6304
**Neuromast width ( *Aulonocara *)**	209	0.88			
SL			1441.915	1, 205	<.0001
Canal			16.9214	1, 205	<.0001
SL x Canal			0.4012	1, 205	0.5272

**Figure 12:**
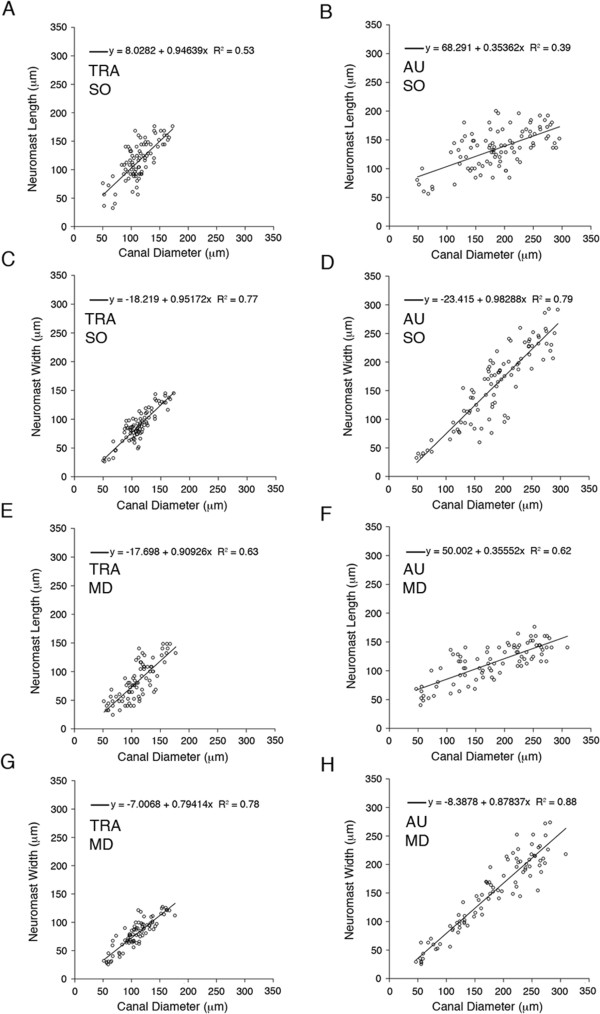
**Relationsihp of neuromast size (length and width) and canal diameter in *Tramitichromis *and *Aulonocara*.** Correlations of neuromast size and canal diameter in the supraorbital **(A, C)** and mandibular **(E, G)** in *Tramitichromis* (TRA) and the supraorbital **(B, D)** and mandibular **(F, H)** in *Aulonocara* (AU). Neuromast length is illustrated in **A**, **E** for *Tramitichromis* and **B**, **F** for *Aulonocara*. Neuromast width is illustrated in **C**, **G** for *Tramitichromis* and **D**, **H** for *Aulonocara*. In both SO and MD canals, neuromast length appears to increase isometrically with canal diameter in *Tramitichromis***(A, E)**, and neuromast length appears to demonstrate a negative allometric trend in *Aulonocara***(B, F)**. Neuromast width appears to increase isometrically with canal diameter in the SO and MD canals in *Tramitichromis***(C, G)** and *Aulonocara***(D, H)**. See Table 5 for results of ANOVA's and ACOVA's.

Analysis was carried out for supraorbital (SO) and mandibular (MD) canals combined in *Tramitichromis* and in *Aulonocara*. SL = Standard length (fish size) in mm. All data were normally distributed. Significance = *P* <0.05. The interaction terms in these ANCOVAs were significant, so the Johnson-Neyman technique was not used. See Figure 12 and Table 2 for ANOVA results and individual regressions.

Finally, a consideration of the correlation between neuromast size and canal diameter also showed some interesting trends (Figure 12, Table 5). In *Tramitichromis*, SO neuromast length (Figure 12A) and width (Figure 12C) both appeared to increase isometrically (slope approximately = 1) with canal diameter. In the MD canal, neuromast length (Figure 12E) appeared to increase isometrically with canal diameter, but neuromast width (Figure 12G) appeared to increase at a slower rate (Table 5). In *Aulonocara*, neuromast width (Figure 12D,H) appeared to increase isometrically with canal diameter in both the SO and MD canals (Table 5)*.* In contrast, neuromast length increased slowly with canal diameter in either the SO or MD canals (Figure 12B,F, Table 5).

**Table 5:** Relationship between neuromast size (length, width) and canal diameter in the supraorbital and mandibular canals

	***Tramitichromis***	***Aulonocara***
**Neuromast length vs. Canal diameter**		
Combined (SO + MD)	y = 0.97x-8.54, R^2^ = 0.50	y = 0.36x + 57.48; R^2^ = 0.47
SO canal	y = 0.95x + 8.0, R^2^ = 0.53	y = 0.35x + 68.29, R^2^ = 0.39
MD canal	y = 0.91x-17.7, R^2^ = 0.63	y = 0.35x + 50.0; R^2^ = 0.62
**Neuromast width vs. Canal diameter**		
Combined (SO + MD)	y = 0.88x-12.6, R^2^ = 0.76	y = 0.92x-14.8, R^2^ = 0.83
SO canal	y = 0.95x-18.2, R^2^ = 0.77	y = 0.98x-23.41, R^2^ = 0.79
MD canal	y = 0.79x-7.0, R^2^ = 0.78	y = 0.87x-8.39, R^2^ = 0.88

Regressions for neuromast length or width (y) versus canal diameter (x), for the supraorbital (SO) and mandibular (MD) canals combined or individually in each species. See also Figure 12.

### Timing of onset of canal enclosure and ossification

**Figure 13:**
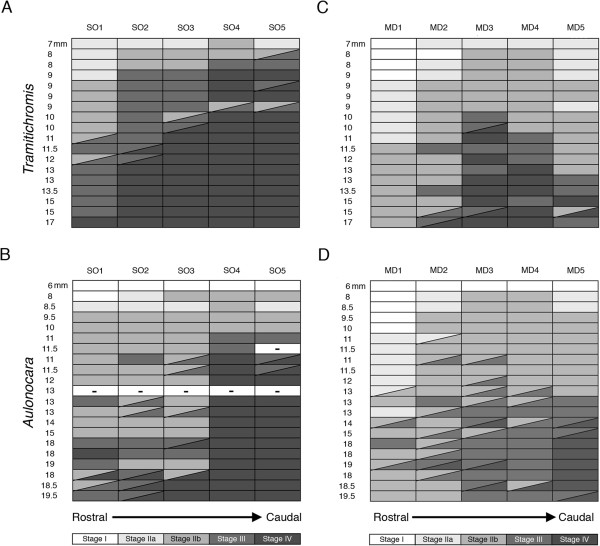
**Ontogenetic trends in order and timing of canal segment morphogenesis in SO and MD canals.** Staging of the development of each canal segment is illustrated for the supraorbital (SO) canal in *Tramitichromis***(A)**, *Aulonocara***(B)**, and the mandibular (MD) canal in *Tramitichromis***(C)** and *Aulonocara***(D)** in larvae and juveniles (6 to 19.5 mm SL). Light to dark grays represent the progression among developmental stages for each canal segment (white = Stage I, darkest gray = Stage IV) on right and left sides, denoted by diagonal lines - see key. Note that in *Tramitichromis* canal segments become enclosed (Stage III) and ossified (Stage IV) at smaller fish sizes than in *Aulonocara* in both the SO and MD canals. In both species, the SO canal shows directionality in development (caudal to rostral) and the MD canal shows weak bidirectionality in *Tramitichromis* only, whereas no pattern was evident in *Aulonocara*.

Fish size at first enclosure (Stage III, Figure 8D,I) and at ossification (Stage IV, Figure 8E,J) for each canal segment within a canal were used to approximate differences in time of onset of these critical stages of canal morphogenesis (Table 6). SO canal segments tended to enclose in a caudal to rostral direction in both species (Figure 13A,B), but the onset of SO segment enclosure tended to occur in larger individuals of *Aulonocara* than of *Tramitichromis*. MD canal segments appeared to enclose bi-directionally, starting with MD3 (Figure 13C,D) in both species, but as in the SO canal, the onset of enclosure occurred in larger individuals of *Aulonocara* than of *Tramitichromis* (Table 6). The MD1 segment was the last of the MD segments to enclose in *Aulonocara* (at approximately 18 mm SL; Figure 7I,K), and had not enclosed in *Tramitichromis* in the largest of individuals examined. Ossification of the SO and MD canal segments was also delayed, with onset in larger individuals of *Aulonocara* than *Tramitichromis*. The order of canal ossification tended to occur in a caudal-to-rostral direction in the SO in both species. The order of ossification among segments in the MD canal could not be discerned in *Aulonocara*, but the order of canal segment ossification was bi-directional in *Tramitichromis* starting with MD3 and progressing both rostrally and caudally (Figure 13).

**Table 6:** Timing and order of canal segment enclosure (Stage III) and ossification (Stage IV)

		**Enclosure (Stage III)**		**Ossification (Stage IV)**	
**Species**	**Canal neuromast**	**Mean fish size**	**Min. canal diameter**	**Mean fish size**	**Min. canal diameter**
*Tramitichromis*	SO1	13.2	83.8	17.0	137.2
sp.	SO2	9.8	86.7	14.0	105.8
	SO3	9.2	83.7	13.3	91.0
	SO4	8.3	100.4	12.0	95.9
	SO5	8.4	98.0	12.1	77.9
	MD1	--*	N/A	--**	---
	MD2	13.7	102.1	17.0	116.9
	MD3	11.8	101.5	13.4	89.7
	MD4	12.4	116.2	14.3	101.4
	MD5	14.5	130.0	15.0	149.7
*Aulonocara*	SO1	17.3	186.6	18	231.4
*stuartgranti*	SO2	15.8	143.0	18.7	225.7
	SO3	14.9	176.8	18.7	227.6
	SO4	11.3	100.4	15.1	120
	SO5	11.1	115.7	15.5	147.2
	MD1	17.3	217.8	--**	---
	MD2	15.0	113.9	19	212.8
	MD3	16.0	112.5	17.2	206.9
	MD4	16.5	166.6	18.5	203.6
	MD5	14.4	167.2	17.7	208.2

Mean fish size and minimum canal diameter at which individual canal segments in the supraorbital (SO) and mandibular (MD) canals in *Tramitichromis* and *Aulonocara* were enclosed (Stage III) and ossified (Stage IV), as derived from histological material. See Figure 2 for definition of stages. Ascending values of mean fish size at enclosure and ossification among segments within a canal were used to infer the order of canal enclosure and ossification within that canal (see text for additional details). *Not enclosed; **Not ossified.

The delay of onset of SO and MD canal segment enclosure (Figures 10 and 13) resulted in canal segment enclosure and ossification at larger diameters in *Aulonocara* than in *Tramitichromis* (Table 6, Figures 8, 13). SO canal segment enclosure started at diameters of >100 μm in *Aulonocara,* but at smaller diameters (<100 μm) in *Tramitichromis*. Subsequent ossification (which occurred after some expansion of canal diameter) started at diameters of up to >200 μm in *Aulonocara*, but at smaller diameters (<150 μm) in *Tramitichromis*. Similarly, MD canal segment enclosure started at diameters of up to >200 μm in *Aulonocara,* but at smaller diameters (<150 μm) in *Tramitichromis*. Subsequent ossification of MD canal segments started at canal diameters of >200 μm in *Aulonocara*, but at smaller diameters (<150 μm) in *Tramitichromis*.

## Discussion

### Heterochrony and the evolution of widened lateral line canals

The cranial lateral line canal systems of *Aulonocara* and *Tramitichromis* contain the same number of canal neuromasts and are characterized by the same pattern of neuromast-centered canal morphogenesis. Nevertheless, the quantitative analysis of canal and neuromast development reported here has demonstrated that the evolution of the widened canal phenotype is the result of heterochrony, and of dissociated heterochrony, in particular. Dissociated heterochrony, defined as a mixture of positive and negative heterochronic shifts (peramorphosis and paedomorphosis of [12]) was illustrated by a higher rate of increase in canal diameter and neuromast width, but a delay in the onset and duration of canal enclosure and ossification in *Aulonocara* relative to *Tramitichromis*. The presence of large canal pores in the widened canals in *Aulonocara* compared to those in *Tramitichromis* is predicted to be the result of either a truncation (earlier offset) or a deceleration in the process of canal roof ossification.

Neuromast width is clearly constrained by canal diameter, but neuromast length is only constrained by the close proximity of adjacent neuromasts, which influences the process of neuromast-centered canal morphogenesis by determining the location, spacing, and size of pores (Figures 2, 7 and 8). The ability of neuromast shape to vary in both developmental and evolutionary time is predicted to have important implications for mechanosensory function (detection of water flows, [59]).

The mechanisms underlying heterochronic change are not revealed by the analysis of developmental patterns. However, it is predicted that the evolution of lateral line canal phenotype is the result of system-wide changes in genetic regulation of neuromast growth (for example, support cell and hair cell differentiation) and/or canal morphogenesis (for example, osteogenesis and remodelling of dermal bone). The correlation between the rate of increase in canal diameter and neuromast width (demonstrated by isometric trend in *Aulonocara*) suggests that there is either a developmental interaction between presumptive canal neuromasts and canal bone (for example, neuromast induction of canal morphogenesis, [60-63]), or a common developmental mechanism guiding the development of both of these tissue types.

### A consideration of modularity in the cranial lateral line system

Modules are defined as parts of developmental or genetic networks, or phenotypes, that compose a hierarchy within an organism [64,65] that can be identified in functional, anatomical, developmental or genetic contexts [7]. Modularity is defined by the interplay between independence among modules and integration among elements within a module [6,53-57,66,67]. The detection of modules allows a more robust picture of morphological evolution [66]. The relationship between modularity and evolution can be revealed by the examination of development at both global and local levels, which may reveal vastly different evolutionary patterns [13]. It has been suggested that the precise relationship between modularity and heterochrony is unknown [67], but, if modularity is required for heterochrony to occur at local scales [13,26], then the detection of local heterochronies within species [13,54,68] may be used to identify potential sites of independence or dissociation, [6] among modules. Furthermore, the ability to respond to demands for evolutionary change [6,69], including those that occur via heterochrony (for example, those shown in this study), will be dependent on the degree of independence among modules.

The nature of the structural hierarchy that defines the cranial lateral line canal system strongly suggests a modular organization, which is defined as follows: 1) individual canal segments, 2) the series of canal segments that compose a lateral line canal (for example, SO1 to SO5, MD1 to SO5), 3) the portions of a canal in different dermal bones composed of one or more canal segments (for example, the portions of the SO canal in the nasal and frontal bones, and the portions of the MD canal in the dentary and anguloarticular bones), 4) each lateral line canal (for example, MD, SO, IO and PO canals), 5) the entire cranial lateral line canal system (all canals), and 6) the entire lateral line canal system (cranial and trunk canals). The superficial neuromasts on the head and trunk appear to comprise at least one additional component (or module) of the lateral line system, which demonstrates developmental trajectories and evolutionary trends different from those of the canal neuromasts [58,70]. The hypothesis that the cranial lateral line canal system is modular is also supported by the stereotyped location of canal neuromasts within individual canal segments (Figure 4), which is the result of neuromast-centered canal morphogenesis (Figures 2, 7 and 8), and the association of different lateral line canals with different dermal bones (Figure 1; [60,71]) that have distinct structural attributes, functional roles, and are subject to different constraints within the skull [27,72,73].

The location of the lateral line canals within dermatocranial bones suggests that the structural (and thus functional) evolution of the lateral line canal system should be constrained by the developmental origins [74,75] and structural and/or functional demands of those dermal bones, and *vice versa*. However, if the morphology of the lateral line canals (and canal neuromasts) is to respond to selection pressures for changes in sensory function (for example, increased sensitivity to water flows that occurs with an evolutionary change from a narrow to a widened phenotype [34,35]), then the canals (and neuromasts) need to be able to evolve independently of the dermal bones in which they are found. For instance, it has recently been shown that mandibular morphology in Lake Malawi cichlids (*Labeotropheus*, *Metriaclima*[76]) can evolve in response to changes in demands on feeding mechanics. This has occurred without a change in the phenotype of the MD lateral line canal (both have narrow canals [47]), but a change in MD canal phenotype (from narrow to widened) has also occurred without substantial changes in mandibular morphology (*Metriaclima* vs. *Aulonocara*, [47]). Thus, it appears that the lateral line canals compose a module that is distinct from (and is independent of) the dermatocranial bones in which they are found.

The SO and MD canals are located in the dorsal bones of the skull and in the mandible, respectively. The nasal and frontal bones in which the SO canal is contained are immobile. In stark contrast, the dentary and anguloarticular bones are mobile and play critical roles in prey acquisition and generation of respiratory flow for gill ventilation, and are thus parts of different functional units in the skull (for example, [69]). Local heterochronies were sought out at several levels within each of the two study species: among lateral line canals (SO vs. MD), among canal portions (for example, the SO portions in the nasal vs. frontal bones), and among canal segments (for example, SO 1 to 5). However, local heterochronies were only found among some of the canal segments within a canal, whereas, the two canal portions of the SO and of the MD canals, and the SO and MD canals themselves did not reveal local heterochronies. It had been predicted that the MD canal would be wider than the SO canal, especially in *Aulonocara*, given its role in detection of benthic prey [36], but this was not the case. Instead, the SO and MD canals demonstrated the same ontogenetic trajectories (developmental rates), suggesting modular integration, but the SO canal was shown to be consistently wider than the MD canal in each of the two species.

It is concluded that the lateral line canals and portions of lateral line canals contained within different dermal bones are influenced more strongly by their canal identity than by the developmental origins and/or structural and functional demands of the different dermal bone(s) in which they are found. These results confirm findings in another study [47] that showed that canal morphology and dermal bones can evolve independently and support the notion that integration at several levels (canals, canal portions, canal segments) maintains the integrity of the lateral line canal system as a single functional unit (module; [66,77]).

### Heterochrony, modularity and the convergent evolution of widened canals

Widened canals have evolved among a small number of diverse families of marine and freshwater fishes [33]. Several groups of mesopelagic (deep-water) marine fishes have generally widened canals (for example, melamphaeids, morids, macrourids; reviewed by [27]), which presumably evolved in response to the need to detect hydrodynamic disturbances in a featureless, three-dimensional environment. In contrast, some benthic feeding freshwater and marine species demonstrate mosaic lateral line phenotypes (narrow and widened phenotypes among different canals in the same individual). Two notable examples are the freshwater cyprinid *Notropis buccatus* (=E*ricymba buccata*, silverjaw minnow, [78]) and marine pleuronectid flatfishes of the genus *Glyptocephalus* (rex sole, witch flounder, [79-81]), both of which are thought to use their lateral line systems to detect benthic invertebrate prey living in sandy substrates, like *Aulonocara*[36]. In *N. buccatus*, the dorsal canals (supraorbital, supratemporal) are narrow and the ventral, or ventrally directed canals (mandibular, preopercular, infraorbital) are widened, with unusual, elongate neuromasts that extend across the canal under bony bridges that represent a reduced canal roof [78], not unlike those in zebrafish (*Danio rerio*[31], unpublished observations). In *Glyptocephalus* species the canals on the eyed (right, functionally dorsal) side are narrow, but those on the blind (left, functionally ventral) side are widened with large diamond-shaped canal neuromasts [79-81]. Evolution of the widened canals in these fishes is likely the result of natural selection for functional modification of a subset (ventral or left, in *N. buccatus* and *Glyptocephalus* species, respectively) of canals via local heterochrony, presumably during the larval stage. In contrast, the overall similarity in morphology among canals and similarity in rates for the SO (dorsal) and MD (ventral) canals in *Aulonocar*a suggests a resistance to local heterochronic shifts in favor of more global evolutionary changes in lateral line canal morphology. *Aulonocara* species inhabit a range of depths (to 70 meters) and often live in caves in Lake Malawi [45,46]. Widened lateral line canals may have initially evolved for enhanced reception of stimuli associated with, for instance, predator avoidance or social interactions in low light environments, and subsequently took on a role in the detection of benthic prey.

The convergent evolution of widened lateral line canals among diverse fish taxa may have occurred in response to a need for enhanced detection of water flows in different behavioral contexts, and thus in response to different selective pressures. The occurrence of all widened canals in several mesopelagic taxa [27], *Aulonocara* (this study, [47]) and Eurasian ruffe, *Gymnocephalus cernuus*[82,83], in contrast to the mosaic canal morphology in other species (*N. buccatus*, *Glyptocephalus*) suggests that the modular organization of the lateral line canal system is manifested differently in different taxa. This draws attention to an unappreciated relationship between modularity and both heterochrony and adaptive evolution.

### Sensory ontogeny, heterochrony, and the life history of fishes

The timing of morphogenesis and onset of function in sensory systems is critical in the life history of fishes (discussed by [32]). It follows that evolutionary change in developmental timing will be important for sensory function in the early life history of fishes, and that such insights are important for linking heterochronic change to adaptive functional evolution. It has been demonstrated that *Tramitichromis* are visual feeders that do not depend on the lateral line system (narrow canals) for prey detection [37]. In contrast, *Aulonocara* use their lateral line system (widened canals) to detect prey, especially in the dark [36,37]. The data presented here have shown that the rate of increase in canal diameter is faster, but that morphogenesis of the lateral line canals is somewhat delayed in *Aulonocara* relative to *Tramitichromis*. As a result, enclosure and ossification of canal segments occur at larger canal diameters in *Aulonocara*. For example, in* Aulonocara* larvae of 15 mm SL (after the normal time of release from the mother’s mouth), the MD canal is already 1.4x wider than it is in *Tramitichromis*. Larger diameter canals presumably function at a higher Reynolds Number (*Re*, the ratio of inertial to viscous forces), which would facilitate displacement of the cupulae (stimulation) of canal neuromasts in response to water movements thus enhancing the probability of prey detection. Evolutionary change in lateral line phenotype accomplished via simple changes in rates of increase in canal diameter and neuromast size and a delay of the onset of canal morphogenesis may be particularly well-suited for the life history of these mouth brooders, in which larvae (with a prolonged yolk-sac stage) reside in the mother’s mouth without having to feed for several weeks post-hatch while the lateral line canals start to develop. A comparison of development of narrow and widened canal systems in non-mouth brooders with free living larvae will reveal more about the relationship among developmental processes, early life history strategies and adaptive morphological evolution in the lateral line system.

## Conclusions

The evolution of widened lateral line canals in *Aulonocara* is the result of dissociated heterochrony-acceleration in the rate of increase of both canal diameter and neuromast size, and delay in the onset of canal morphogenesis relative to *Tramitichromis*. Common rates of increase in canal diameter and neuromast size among canal portions in different dermatocranial bones, among canal segments associated with individual canal neuromasts reflect the absence of local heterochronies and suggest modular integration among canals in both *Aulonocara* and *Tramitichromis*. Thus, canal and neuromast morphology are more strongly influenced by their identities as features of the lateral line system than by the attributes of the dermatocranial bones in which the canals are found. Rate heterochrony manifested during the larval stage ensures that the widened canal phenotype in *Aulonocara*, known to be associated with benthic prey detection in adults, is already present before feeding commences. The lateral line system provides a valuable context for novel analyses of the relationship between developmental processes and the evolution of behaviorally and ecologically relevant phenotypes in fishes.

## Abbreviations

ANCOVA: Analysis of covariance; dpf: Days post-fertilization; IO: Infraorbital canal; L: Liter; MD: Mandibular canal; mm: Millimeters; PO: Preopercular canal; ppt: Parts per thousand; SL: Standard length; SO: Supraorbital canal; μm: Micrometers.

## Competing interests

The authors declare that they have no competing interests.

## Authors’ contributions

NCB and JFW designed the study. NCB collected and analyzed the data. NCB wrote the first draft and both authors revised the manuscript, and approved it for final publication.

## Supplementary Material

Additional file 1: Table S1 Summary of differences in canal diameter and neuromast size (length, width) among canals, canal portions in different bones, and canal segments in *Tramitichromis* and in *Aulonocara*. “=” denotes no difference in size or rate of increase. **Table S2.** ANCOVA for canal diameter and neuromast size (length, width) for SO and MD canal portions in two species. See Table S3 for ANOVA results and regressions. **Table S3.** ANOVA for canal diameter, neuromast size (length, width) in the two portions of the SO and the MD canals. See also Tables S2 and S8. **Table S4.** ANCOVA for canal diameter for five segments in the SO and the MD canals in two species. (see text and Table 3 for additional details, and Table S7 for ANOVA). **Table S5.** ANCOVA for neuromast length for five segments in the SO and the MD canals in two species. (see text and Table 3). See Table S7 for ANOVA results. **Table S6.** ANCOVA for neuromast width for five segments in the SO and the MD canals in two species. (see text and Table 3 for additional details and Table S7 for ANOVA results). **Table S7.** ANOVA for canal diameter, neuromast size (length, width) in the five segments of the SO and the MD canals. See Tables S4-6 and S9 for ANCOVAs. **Table S9.** ANCOVA for canal diameter and neuromast size (length, width) in *Tramitichromis* and *Aulonocara* in the five segments of the SO and the MD canals. ANCOVAs were run for all ten comparisons simultaneously. See Table S7 for ANOVA. Click here for file
