# Supplementary material for: Heterochrony, modularity, and the functional evolution of the mechanosensory lateral line canal system of fishes
Source: EvoDevo. 2014 Jun 5;5:21. doi: 10.1186/2041-9139-5-21 (PMC4066827; doi:10.1186/2041-9139-5-21)
Supplement: Additional file 1: Table S1 — Summary of differences in canal diameter and neuromast size (length, width) among canals, canal portions in different bones, and canal segments in Tramitichromis and in Aulonocara. “=” denotes no difference in size or rate of increase. Table S2. ANCOVA for canal diameter and neuromast size (length, width) for SO and MD canal portions in two species. See Table S3 for ANOVA results and regressions. Table S3. ANOVA for canal diameter, neuromast size (length, width) in the two portions of the SO and the MD canals. See also Tables S2 and S8. Table S4. ANCOVA for canal diameter for five segments in the SO and the MD canals in two species. (see text and Table 3 for additional details, and Table S7 for ANOVA). Table S5. ANCOVA for neuromast length for five segments in the SO and the MD canals in two species. (see text and Table 3). See Table S7 for ANOVA results. Table S6. ANCOVA for neuromast width for five segments in the SO and the MD canals in two species. (see text and Table 3 for additional details and Table S7 for ANOVA results). Table S7. ANOVA for canal diameter, neuromast size (length, width) in the five segments of the SO and the MD canals. See Tables S4-6 and S9 for ANCOVAs. Table S9. ANCOVA for canal diameter and neuromast size (length, width) in Tramitichromis and Aulonocara in the five segments of the SO and the MD canals. ANCOVAs were run for all ten comparisons simultaneously. See Table S7 for ANOVA. [file 2041-9139-5-21-S1.docx]

**Additional Files – Supplementary Tables**

**Table S1 Comparison of module size and development.** Summary of differences in canal diameter and neuromast size (length, width) among canals, canal portions in different bones, and canal segments in *Tramitichromis* and in *Aulonocara*. “=” denotes no difference in size or growth rate.

|  |  | *Tramitichromis* | | |  | *Aulonocara* | | |  |
| --- | --- | --- | --- | --- | --- | --- | --- | --- | --- |
|  |  | **Canal Diameter** | **NM Length** | **NM Width** |  | **Canal Diameter** | **NM Length** | **NM Width** |  |
| **Canals** | |  |  |  |  |  |  |  |  |
|  | **MD vs SO** | SO - 5 μm wider than MD |  |  |  | SO- 11 μm wider than MD |  |  |  |
| **Portions** | |  |  |  |  |  |  |  |  |
|  | **Anguloarticular vs Dentary** | Anguloarticular - 14 μm wider than Dentary | = | = |  | = | Dentary - 10 μm longer than Anguloarticular | Anguloarticular -14 μm longer than Dentary |  |
|  | **Nasal vs Frontal** | Frontal - 8 μm wider than Nasal | Frontal - 33 μm longer than Nasal | Frontal - 13 μm wider than Nasal |  | = | Frontal - 21 μm longer than Nasal | = |  |
| **Segments** | |  |  |  |  |  |  |  |  |
|  | **MD1 vs MD2** | Rate Difference | MD2 - 11 μm longer than MD1 | MD2 - 10 μm wider than MD1 |  | Rate Difference | MD2 - 12 μm longer than MD1 | = |  |
|  | **MD1 vs MD3** | MD3 - 16 μm wider than MD1 | MD3 - 38 μm longer than MD1 | MD3 -13 μm wider than MD1 |  | Rate Difference | MD3 - 33 μm longer than MD1 | = |  |
|  | **MD1 vs MD4** | MD4 - 17 μm wider than MD1 | Rate Difference | MD4 - 13 μm wider than MD1 |  | MD4 - 10 μm wider than MD1 | MD4 - 36 μm longer than MD1 | MD4 - 21 μm wider than MD1 |  |
|  | **MD1 vs MD5** | MD5 - 24 μm wider than MD1 | Rate Difference | MD5 - 11 μm wider than MD1 |  | MD5 - 4 μm wider than MD1 | MD5 - 10 μm longer than MD1 | MD5 - 25 μm wider than MD1 |  |
|  | **MD2 vs MD3** | Rate Difference | MD3 - 27 μm longer than MD2 | = |  | = | MD3 - 21 μm longer than MD2 | = |  |
|  | **MD2 vs MD4** | Rate Difference | MD4 - 33 μm longer than MD2 | = |  | = | MD4 - 24 μm longer than MD2 | Rate Difference |  |
|  | **MD2 vs MD5** | Rate Difference | = | = |  | MD5 - 24 μm wider than MD2 | = | Rate Difference |  |
|  | **MD3 vs MD4** | = | MD4 - 6 μm longer than MD3 | = |  | = | = | = |  |
|  | **MD3 vs MD5** | = | MD3 - 22 μm longer than MD5 | = |  | = | Rate Difference | = |  |
|  | **MD4 vs MD5** | = | MD4 - 29 μm longer than MD5 | = |  | = | MD4 - 26 μm longer than MD5 | = |  |
|  |  |  |  |  |  |  |  |  |  |
|  | **SO1 vs SO2** | SO2 -11 μm wider than SO1 | SO2 - 23 μm longer than SO1 | SO2 - 12 μm wider than SO1 |  | Rate Difference | = | SO2 - 21 μm wider than SO1 |  |
|  | **SO1 vs SO3** | = | SO3 - 28 μm longer than SO1 | SO3 - 14 μm wider than SO1 |  | SO3 - 15 μm wider than SO1 | SO3 - 11 μm longer than SO1 | SO3 - 15 μm longer than SO1 |  |
|  | **SO1 vs SO4** | = | SO4 - 48 μm longer than SO1 | SO4 - 13 μm wider than SO1 |  | SO1 - 28 μm wider than SO4 | SO4 - 40 μm longer than SO1 | = |  |
|  | **SO1 vs SO5** | = | SO5 - 32 μm longer than SO1 | SO5 - 14 μm wider than SO1 |  | = | SO5 - 25 μm longer than SO1 | SO5 - 21 μm longer than SO1 |  |
|  | **SO2 vs SO3** | = | = | = |  | Rate Difference | = | = |  |
|  | **SO2 vs SO4** | = | SO4 - 25 μm longer than SO2 | = |  | Rate Difference | SO4 - 31 μm longer than SO2 | Rate Difference |  |
|  | **SO2 vs SO5** | = | Rate Difference | = |  | SO2 - 34 μm wider than SO5 | SO5 - 16 μm longer than SO2 | = |  |
|  | **SO3 vs SO4** | = | SO4 - 20 μm longer than SO3 | = |  | SO3 - 44 μm wider than SO4 | SO4 - 29 μm longer than SO3 | SO3 - 21 μm wider than SO4 |  |
|  | **SO3 vs SO5** | = | Rate Difference | = |  | SO3 - 17 μm wider than SO5 | SO5 - 14 μm longer than SO3 | = |  |
|  | **SO4 vs SO5** | = | Rate Difference | = |  | SO5 - 27 μm wider than SO4 | SO4 - 14 μm longer than SO5 | SO5 - 26 μm wider than SO4 |  |

**Table S2 ANCOVA for canal diameter and neuromast size (length, width) for portions in the SO and MD canals in two species.** Analysis was carried out for the two SO and the two MD canal portions in *Tramitichromis* and *Aulonocara*. SL = Standard length (fish size) in mm. All data was found to be normal. Significance = *P*<0.05. The Johnson-Neyman technique was used to determine the region of non-significance for fish size for ANCOVAs with significant interaction terms (indicating heterogeneity of slopes, see text and Table 3 for additional details). See Table S3 for ANOVA results and individual regressions.

| **Anguloarticular Bone** | ***N*** | ***R^2^*** | ***F*** | **d.f.** | ***P*** |  | **Frontal Bone** | ***N*** | ***R^2^*** | ***F*** | **d.f.** | ***P*** |
| --- | --- | --- | --- | --- | --- | --- | --- | --- | --- | --- | --- | --- |
| **Canal Diameter** | 37 | 0.89 |  |  |  |  | **Canal Diameter** | 148 | 0.84 |  |  |  |
| Species |  |  | 16.6615 | 1, 33 | 0.0003 |  | Species |  |  | 100.1917 | 1, 144 | <.0001 |
| SL |  |  | 124.8547 | 1, 33 | <.0001 |  | SL |  |  | 290.2352 | 1, 144 | <.0001 |
| Species x SL |  |  | 9.2171 | 1, 33 | 0.0047 |  | Species x SL |  |  | 23.1731 | 1, 144 | <.0001 |
| **Neuromast Length** | 39 | 0.94 |  |  |  |  | **Neuromast Length** | 155 | 0.72 |  |  |  |
| Species |  |  | 416.848 | 1, 35 | <.0001 |  | Species |  |  | 331.794 | 1, 151 | <.0001 |
| SL |  |  | 1.0207 | 1, 35 | 0.3193 |  | SL |  |  | 9.5 | 1, 151 | 0.0024 |
| Species x SL |  |  | 3.9262 | 1, 35 | 0.0554 |  | Species x SL |  |  | 0.2889 | 1, 151 | 0.5917 |
| **Neuromast Width** | 39 | 0.94 |  |  |  |  | **Neuromast Width** | 155 | 0.89 |  |  |  |
| Species |  |  | 41.211 | 1, 35 | <.0001 |  | Species |  |  | 70.9735 | 1, 151 | <.0001 |
| SL |  |  | 263.3391 | 1, 35 | <.0001 |  | SL |  |  | 597.1096 | 1, 151 | <.0001 |
| Species x SL |  |  | 29.7357 | 1, 35 | <.0001 |  | Species x SL |  |  | 50.0405 | 1, 151 | <.0001 |
|  |  |  |  |  |  |  |  |  |  |  |  |  |
| **Dentary Bone** | ***N*** | ***R^2^*** | ***F*** | **d.f.** | ***P*** |  | **Nasal Bone** | ***N*** | ***R^2^*** | ***F*** | **d.f.** | ***P*** |
| **Canal Diameter** | 137 | 0.83 |  |  |  |  | **Canal Diameter** | 36 | 0.91 |  |  |  |
| Species |  |  | 49.0701 | 1, 133 | <.0001 |  | Species |  |  | 31.3619 | 1, 32 | <.0001 |
| SL |  |  | 280.6318 | 1, 133 | <.0001 |  | SL |  |  | 131.9951 | 1, 32 | <.0001 |
| Species x SL |  |  | 35.9174 | 1, 133 | <.0001 |  | Species x SL |  |  | 21.0289 | 1, 32 | <.0001 |
| **Neuromast Length** | 156 | 0.7 |  |  |  |  | **Neuromast Length** | 39 | 0.84 |  |  |  |
| Species |  |  | 269.5093 | 1, 152 | <.0001 |  | Species |  |  | 149.6714 | 1, 35 | <.0001 |
| SL |  |  | 4.6287 | 1, 152 | 0.0330 |  | SL |  |  | 0.0168 | 1, 35 | 0.8977 |
| Species x SL |  |  | 6.2649 | 1, 152 | 0.0134 |  | Species x SL |  |  | 0.612 | 1, 35 | 0.4393 |
| **Neuromast Width** | 156 | 0.91 |  |  |  |  | **Neuromast Width** | 39 | 0.96 |  |  |  |
| Species |  |  | 75.0051 | 1, 152 | <.0001 |  | Species |  |  | 45.6171 | 1, 35 | <.0001 |
| SL |  |  | 723.2492 | 1, 152 | <.0001 |  | SL |  |  | 384.7945 | 1, 35 | <.0001 |
| Species x SL |  |  | 57.7866 | 1, 152 | <.0001 |  | Species x SL |  |  | 41.5879 | 1, 35 | <.0001 |

**Table S3 Results of ANOVA for canal diameter, neuromast size (length, width) in the two portions of the SO and the MD canals.** See Tables S2 and S8 for results of ANCOVAs for these data. All data was found to be normal. Significance level = *P*<0.05.

|  |  | ***N*** | **Regression** | ***R^2^*** | ***P*** |
| --- | --- | --- | --- | --- | --- |
| ***Tramitichromis*** |  |  |  |  |  |
| Canal Diameter |  |  |  |  |  |
| Supraorbital | Nasal | 18 | CD=7.37*SL+25.03 | 0.77 | <.0001 |
|  | Frontal | 72 | CD=8.04*SL+25.19 | 0.71 | <.0001 |
| Mandibular | Dentary | 67 | CD=8.15*SL+13.66 | 0.64 | <.0001 |
|  | Anguloarticular | 18 | CD=9.73*SL+12.25 | 0.8 | <.0001 |
| Neuromast Length | |  |  |  |  |
| Supraorbital | Nasal | 18 | NML=10.24*SL-25.06 | 0.82 | <.0001 |
|  | Frontal | 72 | NML=9.4*SL+17.09 | 0.71 | <.0001 |
| Mandibular | Dentary | 72 | NML=10.09*SL-31.55 | 0.65 | <.0001 |
|  | Anguloarticular | 18 | NML=10.78*SL-47.14 | 0.95 | <.0001 |
| Neuromast Width | |  |  |  |  |
| Supraorbital | Nasal | 18 | NMW=9.26*SL-24.14 | 0.95 | <.0001 |
|  | Frontal | 72 | NMW=9.64*SL-15.12 | 0.92 | <.0001 |
| Mandibular | Dentary | 72 | NMW=9.27*SL-26.35 | 0.87 | <.0001 |
|  | Anguloarticular | 18 | NMW=9.49*SL-26.7 | 0.91 | <.0001 |
| ***Aulonocara*** |  |  |  |  |  |
| Canal Diameter |  |  |  |  |  |
| Supraorbital | Nasal | 18 | CD=17.17*SL-58.70 | 0.86 | <.0001 |
|  | Frontal | 76 | CD=14.37*SL-9.97 | 0.73 | <.0001 |
| Mandibular | Dentary | 70 | CD=17.24*SL-66.46 | 0.78 | <.0001 |
|  | Anguloarticular | 19 | CD=16.99*SL-46.18 | 0.84 | <.0001 |
| Neuromast Length | |  |  |  |  |
| Supraorbital | Nasal | 21 | NML=9.01*SL-9.31 | 0.83 | <.0001 |
|  | Frontal | 83 | NML=8.86*SL+13.42 | 0.72 | <.0001 |
| Mandibular | Dentary | 84 | NML=7.42*SL+8.77 | 0.66 | <.0001 |
|  | Anguloarticular | 21 | NML=8.87*SL-20.54 | 0.91 | <.0001 |
| Neuromast Width | |  |  |  |  |
| Supraorbital | Nasal | 21 | NMW=18.33*SL-103.85 | 0.95 | <.0001 |
|  | Frontal | 83 | NMW=17.49*SL-80.33 | 0.85 | <.0001 |
| Mandibular | Dentary | 84 | NMW=16.58*SL-88.33 | 0.88 | <.0001 |
|  | Anguloarticular | 21 | NMW=19.1*SL-106.97 | 0.93 | <.0001 |

**Table S4 ANCOVA for canal diameter for five segments in the SO and the MD canals in two species.** SL = Standard length (fish size) in mm. All data was found to be normal. Significance = *P*<0.05. The Johnson-Neyman technique was used for ANCOVAs with significant interaction terms (see text and Table 3 for additional details). See Table S7 for ANOVA.

|  | ***N*** | ***R^2^*** | ***F*** | **d.f.** | ***P*-value** |
| --- | --- | --- | --- | --- | --- |
| **Canal Diameter (SO1)** | 36 | 0.91 |  |  |  |
| Species |  |  | 31.3619 | 1, 32 | <.0001 |
| SL |  |  | 131.9951 | 1, 32 | <.0001 |
| Species x SL |  |  | 21.0289 | 1, 32 | <.0001 |
| **Canal Diameter (SO2)** | 36 | 0.93 |  |  |  |
| Species |  |  | 141.967 | 1, 32 | <.0001 |
| SL |  |  | 94.6828 | 1, 32 | <.0001 |
| Species x SL |  |  | 1.5155 | 1, 32 | 0.2273 |
| **Canal Diameter (SO3)** | 38 | 0.92 |  |  |  |
| Species |  |  | 62.255 | 1, 34 | <.0001 |
| SL |  |  | 153.4919 | 1, 34 | <.0001 |
| Species x SL |  |  | 14.775 | 1, 34 | 0.0005 |
| **Canal Diameter (SO4)** | 38 | 0.87 |  |  |  |
| Species |  |  | 5.0403 | 1, 34 | 0.0314 |
| SL |  |  | 124.0516 | 1, 34 | <.0001 |
| Species x SL |  |  | 11.9458 | 1, 34 | 0.0015 |
| **Canal Diameter (SO5)** | 36 | 0.88 |  |  |  |
| Species |  |  | 28.3611 | 1, 32 | <.0001 |
| SL |  |  | 96.411 | 1, 32 | <.0001 |
| Species x SL |  |  | 7.8221 | 1, 32 | 0.0087 |
| **Canal Diameter (MD1)** | 25 | 0.9 |  |  |  |
| Species |  |  | 25.5523 | 1, 21 | <.0001 |
| SL |  |  | 48.5287 | 1, 21 | <.0001 |
| Species x SL |  |  | 0.0344 | 1, 21 | 0.8547 |
| **Canal Diameter (MD2)** | 36 | 0.88 |  |  |  |
| Species |  |  | 23.1422 | 1, 32 | <.0001 |
| SL |  |  | 83.3662 | 1, 32 | <.0001 |
| Species x SL |  |  | 26.1451 | 1, 32 | <.0001 |
| **Canal Diameter (MD3)** | 38 | 0.9 |  |  |  |
| Species |  |  | 12.7718 | 1, 34 | 0.0011 |
| SL |  |  | 155.6798 | 1, 34 | <.0001 |
| Species x SL |  |  | 19.5956 | 1, 34 | <.0001 |
| **Canal Diameter (MD4)** | 38 | 0.84 |  |  |  |
| Species |  |  | 7.7999 | 1, 34 | 0.0085 |
| SL |  |  | 89.8698 | 1, 34 | <.0001 |
| Species x SL |  |  | 7.7 | 1, 34 | 0.0089 |
| **Canal Diameter (MD5)** | 37 | 0.89 |  |  |  |
| Species |  |  | 16.6615 | 1, 33 | 0.0003 |
| SL |  |  | 124.8547 | 1, 33 | <.0001 |
| Species x SL |  |  | 9.2171 | 1, 33 | 0.0047 |

**Table S5 ANCOVA for neuromast length for five segments in the SO and the MD canals in two species.** SL = Standard length (fish size) in mm. All data was found to be normal. Significance = *P*<0.05. The Johnson-Neyman technique was used for ANCOVAs with significant interaction terms (see text and Table 3 for additional details). See Table S7 for ANOVA results.

|  | ***N*** | ***R^2^*** | ***F*** | **d.f.** | ***P*** |
| --- | --- | --- | --- | --- | --- |
| **Neuromast Length (SO1)** | 39 | 0.84 |  |  |  |
| Species |  |  | 0.0168 | 1, 35 | 0.8977 |
| SL |  |  | 149.6714 | 1, 35 | <.0001 |
| Species x SL |  |  | 0.612 | 1, 35 | 0.4393 |
| **Neuromast Length (SO2)** | 39 | 0.77 |  |  |  |
| Species |  |  | 3.9288 | 1, 35 | 0.0554 |
| SL |  |  | 97.1448 | 1, 35 | <.0001 |
| Species x SL |  |  | 0.0014 | 1, 35 | 0.9707 |
| **Neuromast Length (SO3)** | 39 | 0.83 |  |  |  |
| Species |  |  | 8.3829 | 1, 35 | 0.0065 |
| SL |  |  | 133.9754 | 1, 35 | <.0001 |
| Species x SL |  |  | 0.491 | 1, 35 | 0.4881 |
| **Neuromast Length (SO4)** | 39 | 0.81 |  |  |  |
| Species |  |  | 1.0117 | 1, 35 | 0.3214 |
| SL |  |  | 112.0848 | 1, 35 | <.0001 |
| Species x SL |  |  | 1.2282 | 1, 35 | 0.2753 |
| **Neuromast Length (SO5)** | 38 | 0.84 |  |  |  |
| Species |  |  | 2.9658 | 1, 34 | 0.0941 |
| SL |  |  | 160.7478 | 1, 34 | <.0001 |
| Species x SL |  |  | 8.7741 | 1, 34 | 0.0055 |
| **Neuromast Length (MD1)** | 39 | 0.91 |  |  |  |
| Species |  |  | 13.6871 | 1, 35 | 0.0007 |
| SL |  |  | 237.3343 | 1, 35 | <.0001 |
| Species x SL |  |  | 1.0462 | 1, 35 | 0.3134 |
| **Neuromast Length (MD2)** | 39 | 0.91 |  |  |  |
| Species |  |  | 7.865 | 1, 35 | 0.0082 |
| SL |  |  | 257.7994 | 1, 35 | <.0001 |
| Species x SL |  |  | 3.5741 | 1, 35 | 0.0670 |
| **Neuromast Length (MD3)** | 39 | 0.86 |  |  |  |
| Species |  |  | 2.9032 | 1, 35 | 0.0973 |
| SL |  |  | 174.7997 | 1, 35 | <.0001 |
| Species x SL |  |  | 9.9633 | 1, 35 | 0.0033 |
| **Neuromast Length (MD4)** | 39 | 0.83 |  |  |  |
| Species |  |  | 0.0016 | 1, 35 | 0.9688 |
| SL |  |  | 140.3288 | 1, 35 | <.0001 |
| Species x SL |  |  | 4.7219 | 1, 35 | 0.0366 |
| **Neuromast Length (MD5)** | 39 | 0.94 |  |  |  |
| Species |  |  | 1.0207 | 1, 35 | 0.3193 |
| SL |  |  | 416.848 | 1, 35 | <.0001 |
| Species x SL |  |  | 3.9262 | 1, 35 | 0.0554 |

**Table S6 ANCOVA for neuromast width for five segments in the SO and the MD canals in two species.** SL = Standard length (fish size) in mm. All data was found to be normal. Significance = *P*<0.05. The Johnson-Neyman technique was used for ANCOVAs with significant interaction terms (see text and Table 3 for additional details). See Table S7 for ANOVA results.

|  | ***N*** | ***R^2^*** | ***F*** | **d.f.** | ***P*** |
| --- | --- | --- | --- | --- | --- |
| **Neuromast Width (SO1)** | 39 | 0.96 |  |  |  |
| Species |  |  | 45.6171 | 1, 35 | <.0001 |
| SL |  |  | 38.7945 | 1, 35 | <.0001 |
| Species x SL |  |  | 41.5879 | 1, 35 | <.0001 |
| **Neuromast Width (SO2)** | 39 | 0.93 |  |  |  |
| Species |  |  | 33.8073 | 1, 35 | <.0001 |
| SL |  |  | 198.671 | 1, 35 | <.0001 |
| Species x SL |  |  | 25.9088 | 1, 35 | <.0001 |
| **Neuromast Width (SO3)** | 39 | 0.9 |  |  |  |
| Species |  |  | 20.8914 | 1, 35 | <.0001 |
| SL |  |  | 158.7736 | 1, 35 | <.0001 |
| Species x SL |  |  | 12.584 | 1, 35 | 0.0011 |
| **Neuromast Width (SO4)** | 39 | 0.88 |  |  |  |
| Species |  |  | 5.8294 | 1, 35 | 0.0211 |
| SL |  |  | 149.4206 | 1, 35 | <.0001 |
| Species x SL |  |  | 7.6145 | 1, 35 | 0.0091 |
| **Neuromast Width (SO5)** | 38 | 0.91 |  |  |  |
| Species |  |  | 25.3347 | 1, 34 | <.0001 |
| SL |  |  | 167.5909 | 1, 34 | <.0001 |
| Species x SL |  |  | 13.0175 | 1, 34 | 0.0010 |
| **Neuromast Width (MD1)** | 39 | 0.91 |  |  |  |
| Species |  |  | 18.8761 | 1, 35 | 0.0001 |
| SL |  |  | 174.5651 | 1, 35 | <.0001 |
| Species x SL |  |  | 13.5728 | 1, 35 | 0.0008 |
| **Neuromast Width (MD2)** | 39 | 0.95 |  |  |  |
| Species |  |  | 27.5121 | 1, 35 | <.0001 |
| SL |  |  | 313.908 | 1, 35 | <.0001 |
| Species x SL |  |  | 19.3158 | 1, 35 | <.0001 |
| **Neuromast Width (MD3)** | 39 | 0.91 |  |  |  |
| Species |  |  | 20.5418 | 1, 35 | <.0001 |
| SL |  |  | 177.1833 | 1, 35 | <.0001 |
| Species x SL |  |  | 13.3675 | 1, 35 | 0.0008 |
| **Neuromast Width (MD4)** | 39 | 0.93 |  |  |  |
| Species |  |  | 23.3346 | 1, 35 | <.0001 |
| SL |  |  | 225.8952 | 1, 35 | <.0001 |
| Species x SL |  |  | 23.337 | 1, 35 | <.0001 |
| **Neuromast Width (MD5)** | 39 | 0.94 |  |  |  |
| Species |  |  | 41.211 | 1, 35 | <.0001 |
| SL |  |  | 263.3391 | 1, 35 | <.0001 |
| Species x SL |  |  | 29.7357 | 1, 35 | <.0001 |

**Table S7 Results of ANOVA for canal diameter, neuromast size (length, width) in the five segments of the SO and the MD canals.** All data was found to be normal. Significance level = *P*<0.05. See Tables S4-6 and S9 for results of ANCOVAs for these data.

|  | **NM** | ***N*** | **Regression** | ***R2*** | ***P*** |  |  | **NM** | ***N*** | **Regression** | ***R2*** | ***P*** |
| --- | --- | --- | --- | --- | --- | --- | --- | --- | --- | --- | --- | --- |
| ***Tramitichromis*** |  |  |  |  |  |  | ***Aulonocara*** |  |  |  |  |  |
| Canal Diam. |  |  |  |  |  |  | Canal Diam. |  |  |  |  |  |
| Supraorbital | SO1 | 18 | Y=7.37*X+25.03 | 0.77 | <.0001 |  | Supraorbital | SO1 | 18 | Y=17.17*X-58.70 | 0.86 | <.0001 |
|  | SO2 | 18 | Y=7.70*X+32.22 | 0.74 | <.0001 |  |  | SO2 | 18 | Y=9.93*X+76.57 | 0.77 | <.0001 |
|  | SO3 | 18 | Y=8.95*X+14.6 | 0.77 | <.0001 |  |  | SO3 | 20 | Y=17.00*X-30.55 | 0.88 | <.0001 |
|  | SO4 | 18 | Y=7.82*X+26.74 | 0.78 | <.0001 |  |  | SO4 | 20 | Y=14.87*X-45.54 | 0.85 | <.0001 |
|  | SO5 | 18 | Y=7.69*X+27.22 | 0.6 | 0.0002 |  |  | SO5 | 18 | Y=13.81*X-10.26 | 0.85 | <.0001 |
| Mandibular | MD1 | 14 | Y=9.56*X-17.85 | 0.88 | <.0001 |  | Mandibular | MD1 | 11 | Y=10.08*X+22.24 | 0.58 | 0.0062 |
|  | MD2 | 17 | Y=5.06*X+41.7 | 0.61 | 0.0002 |  |  | MD2 | 19 | Y=17.95*X-80.27 | 0.85 | <.0001 |
|  | MD3 | 18 | Y=9.94*X+2.42 | 0.79 | <.0001 |  |  | MD3 | 20 | Y=20.87*X-104.05 | 0.88 | <.0001 |
|  | MD4 | 18 | Y=9.86*X+3.95 | 0.84 | <.0001 |  |  | MD4 | 20 | Y=18.02*X-70.17 | 0.78 | <.0001 |
|  | MD5 | 18 | Y=9.73*X+12.25 | 0.8 | <.0001 |  |  | MD5 | 19 | Y=16.99*X-46.18 | 0.84 | <.0001 |
| NM Length |  |  |  |  |  |  | NM Length |  |  |  |  |  |
| Supraorbital | SO1 | 18 | Y=10.24*X-25.06 | 0.82 | <.0001 |  | Supraorbital | SO1 | 21 | Y=9.01*X-9.31 | 0.83 | <.0001 |
|  | SO2 | 18 | Y=8.83*X+13.67 | 0.75 | <.0001 |  |  | SO2 | 21 | Y=8.90*X+1.04 | 0.77 | <.0001 |
|  | SO3 | 18 | Y=7.93*X+28.82 | 0.84 | <.0001 |  |  | SO3 | 21 | Y=8.95*X+2.31 | 0.82 | <.0001 |
|  | SO4 | 18 | Y=7.56*X+52.93 | 0.81 | <.0001 |  |  | SO4 | 21 | Y=9.32*X+25.96 | 0.81 | <.0001 |
|  | SO5 | 18 | Y=13.30*X-27.07 | 0.89 | <.0001 |  |  | SO5 | 20 | Y=8.26*X+24.94 | 0.77 | <.0001 |
| Mandibular | MD1 | 18 | Y=8.50*X-37.07 | 0.89 | <.0001 |  | Mandibular | MD1 | 21 | Y=7.44*X-11.42 | 0.88 | <.0001 |
|  | MD2 | 18 | Y=10.22*X-45.16 | 0.94 | <.0001 |  |  | MD2 | 21 | Y=8.07*X-8.17 | 0.86 | <.0001 |
|  | MD3 | 18 | Y=10.05*X-16.57 | 0.95 | <.0001 |  |  | MD3 | 21 | Y=6.18*X+37.84 | 0.74 | <.0001 |
|  | MD4 | 18 | Y=11.59*X-27.40 | 0.91 | <.0001 |  |  | MD4 | 21 | Y=7.99*X+16.84 | 0.74 | <.0001 |
|  | MD5 | 18 | Y=10.78*X-47.14 | 0.95 | <.0001 |  |  | MD5 | 21 | Y=8.87*X-20.54 | 0.91 | <.0001 |
| NM Width |  |  |  |  |  |  | NM Width |  |  |  |  |  |
| Supraorbital | SO1 | 18 | Y=9.26*X-24.14 | 0.95 | <.0001 |  | Supraorbital | SO1 | 21 | Y=18.33*X-103.85 | 0.95 | <.0001 |
|  | SO2 | 18 | Y=9.14*X-10.85 | 0.91 | <.0001 |  |  | SO2 | 21 | Y=19.48*X-98.41 | 0.91 | <.0001 |
|  | SO3 | 18 | Y=9.80*X-16.06 | 0.93 | <.0001 |  |  | SO3 | 21 | Y=17.49*X-77.42 | 0.87 | <.0001 |
|  | SO4 | 18 | Y=9.43*X-12.74 | 0.92 | <.0001 |  |  | SO4 | 21 | Y=14.93*X-64.18 | 0.85 | <.0001 |
|  | SO5 | 18 | Y=10.18*X-20.81 | 0.92 | <.0001 |  |  | SO5 | 20 | Y=18.05*X-80.57 | 0.88 | <.0001 |
| Mandibular | MD1 | 18 | Y=9.05*X-33.00 | 0.92 | <.0001 |  | Mandibular | MD1 | 21 | Y=16.05*X-91.44 | 0.88 | <.0001 |
|  | MD2 | 18 | Y=8.98*X-21.87 | 0.91 | <.0001 |  |  | MD2 | 21 | Y=14.91*X-71.06 | 0.94 | <.0001 |
|  | MD3 | 18 | Y=9.20*X-22.05 | 0.9 | <.0001 |  |  | MD3 | 21 | Y=16.18*X-78.85 | 0.88 | <.0001 |
|  | MD4 | 18 | Y=9.85*X-28.50 | 0.92 | <.0001 |  |  | MD4 | 21 | Y=19.19*X-111.97 | 0.91 | <.0001 |
|  | MD5 | 18 | Y=9.49*X-26.70 | 0.91 | <.0001 |  |  | MD5 | 21 | Y=19.10*X-106.97 | 0.93 | <.0001 |

**Table S8 ANCOVA for canal diameter and neuromast size (length, width) in *Tramitichromis* and *Aulonocara* in the two portions of the SO and the MD canals.** Analysis was carried out for SO and MD canals in *Tramitichromis* and in *Aulonocara*. SL = Standard length (fish size) in mm. All data was found to be normal. Significance = *P*<0.05. See Table S3 for ANOVA results.

|  | *N* | *R^2^* | *F* | d.f. | *P*-value |  |  | *N* | *R^2^* | *F* | d.f. | *P*-value |
| --- | --- | --- | --- | --- | --- | --- | --- | --- | --- | --- | --- | --- |
| *Tramitichromis* |  |  |  |  |  |  | *Aulonocara* |  |  |  |  |  |
| Canal Diameter |  |  |  |  |  |  | Canal Diameter |  |  |  |  |  |
| Supraorbital | 90 | 0.73 |  |  |  |  | Supraorbital | 94 | 0.76 |  |  |  |
| SL |  |  | 135.687 | 1, 86 | <.0001 |  | SL |  |  | 180.104 | 1, 90 | <.0001 |
| Portion |  |  | 4.328 | 1, 86 | 0.0398 |  | Portion |  |  | 1.554 | 1, 90 | 0.2158 |
| SL x Portion |  |  | 0.254 | 1, 86 | 0.6154 |  | SL x Portion |  |  | 1.415 | 1, 90 | 0.2374 |
| Mandibular | 85 | 0.69 |  |  |  |  | Mandibular | 89 | 0.79 |  |  |  |
| SL |  |  | 128.762 | 1, 81 | <.0001 |  | SL |  |  | 197.919 | 1, 85 | <.0001 |
| Portion |  |  | 14.400 | 1, 81 | 0.0003 |  | Portion |  |  | 3.869 | 1, 85 | 0.0525 |
| SL x Portion |  |  | 0.997 | 1, 81 | 0.3210 |  | SL x Portion |  |  | 0.011 | 1, 85 | 0.9176 |
| NM Length |  |  |  |  |  |  | NM Length |  |  |  |  |  |
| Supraorbital | 90 | 0.78 |  |  |  |  | Supraorbital | 104 | 0.75 |  |  |  |
| SL |  |  | 161.595 | 1, 86 | <.0001 |  | SL |  |  | 185.660 | 1, 100 | <.0001 |
| Portion |  |  | 60.310 | 1, 86 | <.0001 |  | Portion |  |  | 16.746 | 1, 100 | <.0001 |
| SL x Portion |  |  | 0.291 | 1, 86 | 0.5908 |  | SL x Portion |  |  | 0.012 | 1, 100 | 0.9138 |
| Mandibular | 90 | 0.7 |  |  |  |  | Mandibular | 105 | 0.71 |  |  |  |
| SL |  |  | 134.989 | 1, 86 | <.0001 |  | SL |  |  | 175.935 | 1, 101 | <.0001 |
| Portion |  |  | 2.566 | 1, 86 | 0.1129 |  | Portion |  |  | 4.441 | 1, 101 | 0.0376 |
| SL x Portion |  |  | 0.149 | 1, 86 | 0.7005 |  | SL x Portion |  |  | 1.403 | 1, 101 | 0.2390 |
| NM Width |  |  |  |  |  |  | NM Width |  |  |  |  |  |
| Supraorbital | 90 | 0.93 |  |  |  |  | Supraorbital | 104 | 0.87 |  |  |  |
| SL |  |  | 650.221 | 1, 86 | <.0001 |  | SL |  |  | 451.183 | 1, 100 | <.0001 |
| Portion |  |  | 42.293 | 1, 86 | <.0001 |  | Portion |  |  | 3.564 | 1, 100 | 0.0619 |
| SL x Portion |  |  | 0.259 | 1, 86 | 0.6121 |  | SL x Portion |  |  | 0.249 | 1, 100 | 0.6192 |
| Mandibular | 90 | 0.88 |  |  |  |  | Mandibular | 105 | 0.89 |  |  |  |
| SL |  |  | 417.114 | 1, 86 | <.0001 |  | SL |  |  | 584.364 | 1, 101 | <.0001 |
| Portion |  |  | 0.687 | 1, 86 | 0.4094 |  | Portion |  |  | 6.589 | 1, 101 | 0.0117 |
| SL x Portion |  |  | 0.057 | 1, 86 | 0.8121 |  | SL x Portion |  |  | 2.913 | 1, 101 | 0.0910 |

**Table S9 ANCOVA for canal diameter and neuromast size (length, width) in *Tramitichromis* and *Aulonocara* in the five segments of the SO and the MD canals.** SL = Standard length (fish size) in mm. All data was found to be normal. Significance = *P*<0.05.

ANCOVAs were run for all comparisons simultaneously (10 comparisons). SL (Standard Length) = fish size in mm. Significance level = *P*<0.05. All data normal. See Table S7 for ANOVA results.

|  | ***N*** | ***R^2^*** | ***F*** | **d.f.** | ***P*-value** |  |  | ***N*** | ***R^2^*** | ***F*** | **d.f.** | ***P*-value** |
| --- | --- | --- | --- | --- | --- | --- | --- | --- | --- | --- | --- | --- |
| ***Tramitichromis*** |  |  |  |  |  |  | ***Aulonocara*** |  |  |  |  |  |
| Canal Diameter |  |  |  |  |  |  | Canal Diameter |  |  |  |  |  |
| SO Canal | 90 | 0.73 |  |  |  |  | SO Canal | 94 | 0.87 |  |  |  |
| SL |  |  | 213.131 | 1, 80 | <.0001 |  | SL |  |  | 463.8033 | 1, 84 | <.0001 |
| NM # |  |  | 1.3842 | 4, 80 | 0.2469 |  | NM # |  |  | 17.1166 | 4, 84 | <.0001 |
| SL x NM # |  |  | 0.2503 | 4, 80 | 0.9087 |  | SL x NM # |  |  | 3.5419 | 4, 84 | 0.0101 |
| MD Canal | 85 | 0.82 |  |  |  |  | MD Canal | 89 | 0.83 |  |  |  |
| SL |  |  | 278.8339 | 1, 75 | <.0001 |  | SL |  |  | 268.5122 | 1, 79 | <.0001 |
| NM # |  |  | 16.1318 | 4, 75 | <.0001 |  | NM # |  |  | 1.9319 | 4, 79 | 0.1133 |
| SL x NM # |  |  | 3.3744 | 4, 75 | 0.0136 |  | SL x NM # |  |  | 1.9913 | 4, 79 | 0.1039 |
| Neuromast Length |  |  |  |  |  |  | Neuromast Length |  |  |  |  |  |
| SO Canal | 90 | 0.87 |  |  |  |  | SO Canal | 104 | 0.83 |  |  |  |
| SL |  |  | 381.0863 | 1, 80 | <.0001 |  | SL |  |  | 381.7456 | 1, 94 | <.0001 |
| NM # |  |  | 33.744 | 4, 80 | <.0001 |  | NM # |  |  | 15.2551 | 4, 94 | <.0001 |
| SL x NM # |  |  | 4.5017 | 4, 80 | 0.0025 |  | SL x NM # |  |  | 0.1447 | 4, 94 | 0.9649 |
| MD Canal | 90 | 0.95 |  |  |  |  | MD Canal | 105 | 0.86 |  |  |  |
| SL |  |  | 1037.479 | 1, 80 | <.0001 |  | SL |  |  | 457.451 | 1, 95 | <.0001 |
| NM # |  |  | 91.0047 | 4, 80 | <.0001 |  | NM # |  |  | 24.3385 | 4, 95 | <.0001 |
| SL x NM # |  |  | 2.5698 | 4, 80 | 0.0440 |  | SL x NM # |  |  | 1.5353 | 4, 95 | 0.1982 |
| Neuromast Width |  |  |  |  |  |  | Neuromast Width |  |  |  |  |  |
| SO Canal | 90 | 0.93 |  |  |  |  | SO Canal | 104 | 0.9 |  |  |  |
| SL |  |  | 992.2433 | 1, 80 | <.0001 |  | SL |  |  | 780.5842 | 1, 94 | <.0001 |
| NM # |  |  | 10.26 | 4, 80 | <.0001 |  | NM # |  |  | 4.711 | 4, 94 | 0.0016 |
| SL x NM # |  |  | 0.3918 | 4, 80 | 0.8139 |  | SL x NM # |  |  | 1.433 | 4, 94 | 0.2292 |
| MD Canal | 90 | 0.91 |  |  |  |  | MD Canal | 105 | 0.91 |  |  |  |
| SL |  |  | 822.0206 | 1, 80 | <.0001 |  | SL |  |  | 932.6354 | 1, 95 | <.0001 |
| NM # |  |  | 7.496 | 4, 80 | <.0001 |  | NM # |  |  | 4.6038 | 4, 95 | 0.0019 |
| SL x NM # |  |  | 0.2433 | 4, 80 | 0.9129 |  | SL x NM # |  |  | 2.4152 | 4, 95 | 0.0541 |
